# SEC61B regulates calcium flux and platelet hyperreactivity in diabetes

**DOI:** 10.1172/JCI184597

**Published:** 2025-08-15

**Authors:** Yvonne X. Kong, Rajan Rehan, Cesar L. Moreno, Søren Madsen, Yunwei Zhang, Huiwen Zhao, Miao Qi, Callum B. Houlahan, Siân P. Cartland, Declan Robertshaw, Vincent Trang, Frederick Jun Liang Ong, Michael Liu, Edward Cheng, Imala Alwis, Alexander Dupuy, Michelle Cielesh, Kristen C. Cooke, Meg Potter, Jacqueline Stöckli, Grant Morahan, Maggie L. Kalev-Zylinska, Matthew T. Rondina, Sol Schulman, Jean Y. H. Yang, G. Gregory Neely, Simone M. Schoenwaelder, Shaun P. Jackson, David E. James, Mary M. Kavurma, Samantha L. Hocking, Stephen M. Twigg, James C. Weaver, Mark Larance, Freda H. Passam

**Affiliations:** 1 Charles Perkins Centre, University of Sydney, Sydney, New South Wales, Australia; 2 Institute of Haematology, Royal Prince Alfred Hospital, Sydney, New South Wales, Australia.; 3 Central Clinical School, Faculty of Medicine and Health, University of Sydney, Sydney, New South Wales, Australia.; 4 Heart Research Institute, University of Sydney, Sydney, New South Wales, Australia.; 5 Department of Cardiology, Royal Prince Alfred Hospital, Sydney, New South Wales, Australia.; 6 The Dr. John and Anne Chong Lab for Functional Genomics, University of Sydney, Sydney, New South Wales, Australia.; 7 School of Life and Environmental Sciences, University of Sydney, Sydney, New South Wales, Australia.; 8 School of Mathematics and Statistics, The University of Sydney, Sydney, New South Wales, Australia.; 9 Sydney Precision Data Science, The University of Sydney, Sydney, New South Wales, Australia.; 10 School of Mathematics, Statistics, Chemistry and Physics, Murdoch University, Perth, Western Australia, Australia.; 11 School of Medical Sciences, University of Sydney, Sydney, New South Wales, Australia; 12 Centre for Peripheral Artery Disease, Heart Research institute, Sydney, New South Wales, Australia; 13 School of Biomedical Engineering, Faculty of Engineering, University of Sydney, Sydney, New South Wales Australia.; 14 Centre for Diabetes Research, Harry Perkins Institute of Medical Research, Nedlands, Western Australia, Australia.; 15 Australian Centre for Accelerating Diabetes Innovations, Melbourne Medical School, The University of Melbourne, Parkville, Victoria, Australia.; 16 Department of Molecular Medicine and Pathology, University of Auckland, Auckland, New Zealand.; 17 Department of Pathology and Laboratory Medicine, Haematology Laboratory, Auckland City Hospital, Auckland, New Zealand.; 18 Molecular Medicine Program, Department of Internal Medicine, and Division of Hematology and Hematologic Malignancies, University of Utah, Salt Lake City, Utah, USA.; 19 Division of Hemostasis and Thrombosis, Beth Israel Deaconess Medical Center and Harvard Medical School, Boston, Massachusetts, USA.; 20 Department of Endocrinology, Royal Prince Alfred Hospital, Sydney, New South Wales, Australia.

**Keywords:** Cardiology, Cell biology, Hematology, Calcium channels, Platelets, Proteomics

## Abstract

Platelet hyperreactivity increases the risk of cardiovascular thrombosis in diabetes and failure of antiplatelet drug therapies. Elevated basal and agonist-induced calcium flux is a fundamental cause of platelet hyperreactivity in diabetes; however, the mechanisms responsible for this remain largely unknown. Using a high-sensitivity, unbiased proteomic platform, we consistently detected over 2,400 intracellular proteins and identified proteins that were differentially released by platelets in type 2 diabetes. We identified that SEC61 translocon subunit β (SEC61B) was increased in platelets from humans and mice with hyperglycemia and in megakaryocytes from mice with hyperglycemia. SEC61 is known to act as an endoplasmic reticulum (ER) calcium leak channel in nucleated cells. Using HEK293 cells, we showed that SEC61B overexpression increased calcium flux into the cytosol and decreased protein synthesis. Concordantly, platelets in hyperglycemic mice mobilized more calcium and had decreased protein synthesis. Platelets in both humans and mice with hyperglycemia had increased ER stress. ER stress induced the expression of platelet SEC61B and increased cytosolic calcium. Inhibition of SEC61 with anisomycin decreased platelet calcium flux and inhibited platelet aggregation in vitro and in vivo. These studies demonstrate the existence of a mechanism whereby ER stress–induced upregulation of platelet SEC61B leads to increased cytosolic calcium, potentially contributing to platelet hyperreactivity in diabetes.

## Abstract 2

This study identifies SEC61 as a novel endoplasmic reticulum calcium leak channel in platelets. Upregulation of SEC61B contributes to platelet hyperreactivity in diabetes.

## Graphical abstract

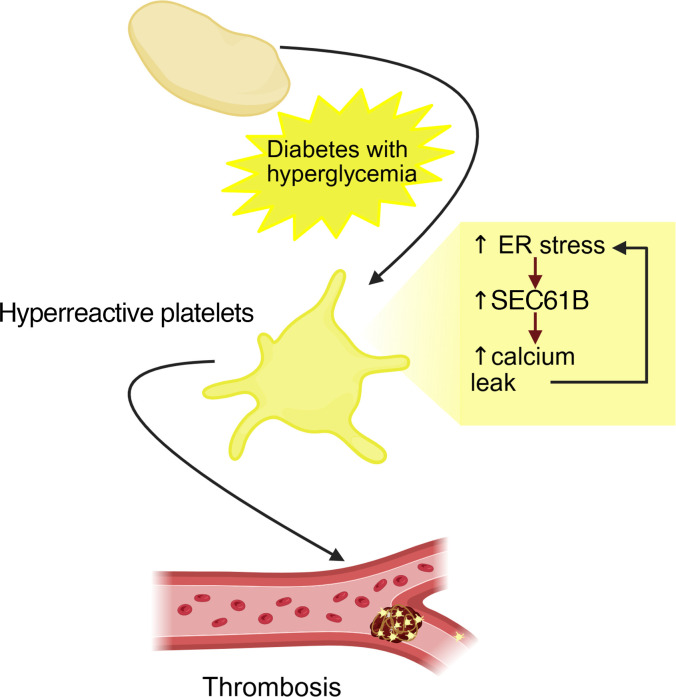

## Introduction

Platelet hyperreactivity is the increased propensity of platelets to activate and secrete their content in response to various stimuli, and it is common in individuals with diabetes mellitus (DM) (1–4). Clinically, this translates into a higher risk of cardiovascular events, such as coronary artery disease, and impaired effectiveness of antiplatelet agents routinely used for treatment (1, 2). Platelet hyperreactivity in diabetes is multifactorial (2). Hyperglycemia increases platelet reactivity by nonenzymatic glycation of platelet membrane glycoproteins and by producing glycated LDLs that promote the formation of reactive oxygen species and activation of protein kinase C (2). Recently, Jain et al. found that diabetic platelets have evidence of endoplasmic reticulum (ER) stress with activation of inositol-requiring enzyme-1 α (IRE1), which is an ER stress sensor (5). However, one of the key features of diabetic platelets is the higher cytosolic calcium under basal conditions and in response to stimuli (3, 4). Understanding the mechanisms of exaggerated platelet calcium responses in diabetes will benefit the development of treatments to combat platelet hyperreactivity.

For platelets to remain in resting state, cytosolic calcium must remain at submicromolar levels (40–100 nM) compared with high submillimolar levels in the ER (dense tubular system) (100–400 µM) and millimolar concentrations in the extracellular space (~2 mM) (6). This is maintained by the sequestration of calcium inside the ER and extrusion of calcium to the extracellular space (6, 7). Calcium is pumped into the ER by sarco/ER Ca2+-ATPases (SERCAs). Platelets contain 2 isoforms: SERCA2b and SERCA3 (8). When platelets are stimulated with various agonists, inositol-1,4,5-trisphosphate (IP3) is produced, which stimulates IP3 receptor channels to release calcium from the ER. This is followed by calcium entry into the cell through plasma membrane calcium channels, a process referred to as store-operated calcium entry (SOCE), leading to downstream calcium signaling pathways and activation of the platelet (7).

Calcium continuously leaks out of the ER through calcium leak channels, a process counteracted by SERCA. In nucleated cells, the SEC61 translocon is the main passive leak channel responsible for approximately 60% of calcium leak from the ER (9–11). The SEC61 translocon functions as a transporter of newly synthesized polypeptides into the ER. When not engaged in protein transport, calcium leaks through SEC61 into the cytosol. Blocking SERCA function, e.g., with thapsigargin (TG), leads to unopposed leak, from the ER through the translocon, and an increase in cytosolic calcium (12). Despite multiple reports on SERCA function in platelets (reviewed in refs. 7, 8, 13), the role of SEC61 in platelets has not been investigated before.

To investigate for potential protein alterations in platelets in diabetes that may explain platelet hyperreactivity, we employed a high-sensitivity proteomic platform that we developed (14). We compared platelets from people with DM, with suspected or known coronary artery disease, and people with similar risk factors without diabetes (non-DM). Our analysis identified that the β subunit of the SEC61 translocon (SEC61B) uniquely correlated with high serum fructosamine, a known measure of glycemic control (15). We subsequently demonstrated SEC61B upregulation in both platelets and megakaryocytes of hyperglycemic mice. SEC61B overexpression was associated with increased calcium leak from the ER into the cytosol, which was inhibited with the SEC61 inhibitor, anisomycin (ANX) (16). We have thus identified SEC61 as a calcium leak channel in platelets and describe SEC61-mediated calcium leak as mechanism of dysregulated calcium flux and platelet hyperreactivity in diabetes.

## Results

We recruited a total of 76 individuals, 42 with type 2 DM and 34 without, from the Cardiology and Endocrinology departments of Royal Prince Alfred Hospital between 2020 and 2021. None of the patients had acute coronary syndromes. All patients provided informed consent and tested negative by COVID-19 PCR before attendance at the hospital, as per local policy at that time.

**Figure 1:**
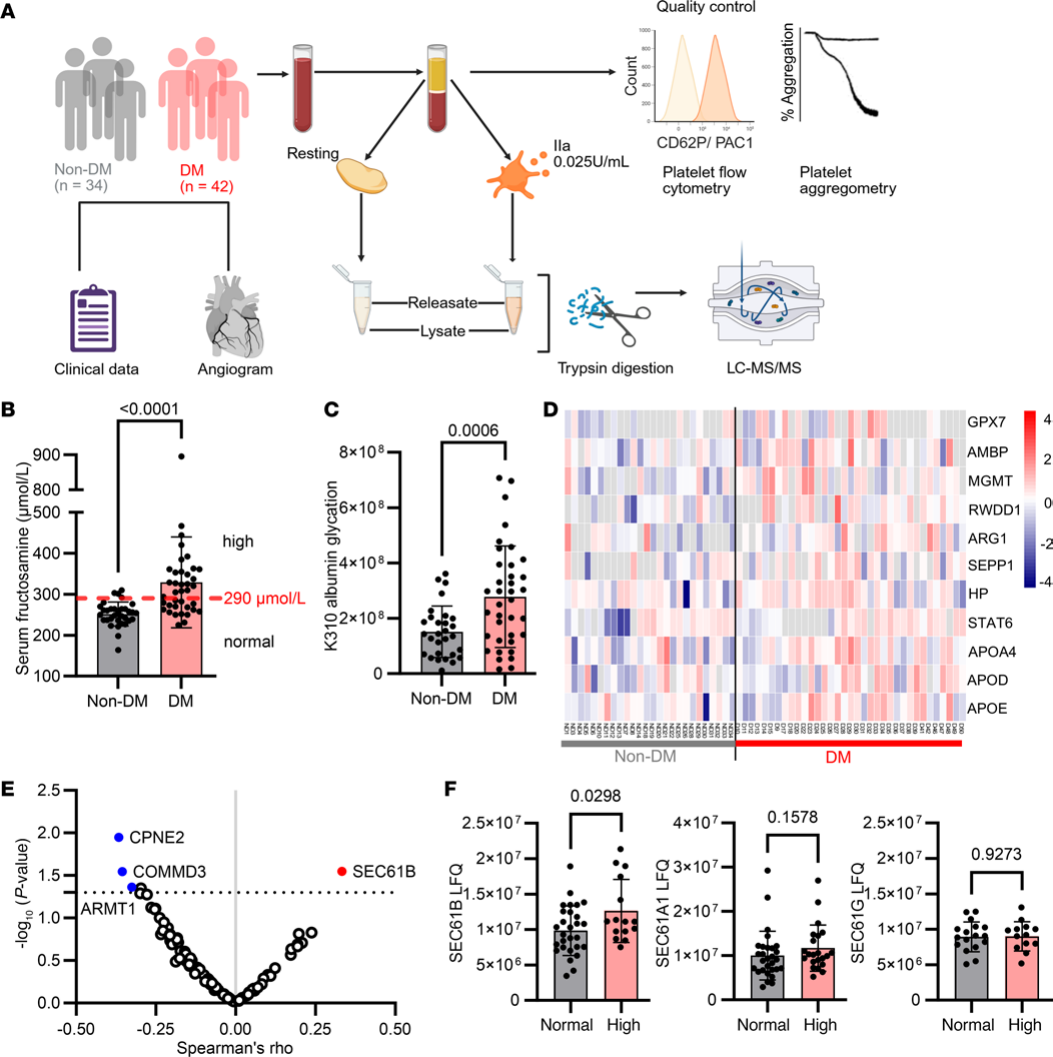
Unbiased, high-sensitivity proteomics of human platelets identifies increased SEC61B in type 2 diabetes. (**A**) Workflow demonstrating collection of clinicolaboratory and coronary angiogram data from patients without (non-DM) and with diabetes (DM); quality check of platelets by flow cytometry; platelet aggregation; and separation of resting and low-dose thrombin-activated platelet intracellular fraction “lysate” and released fraction “releasate” from patients without and with DM. (**B**) Levels of serum fructosamine in patients without (*n* = 33) versus with DM (*n* = 42) (Mann-Whitney test). (**C**) Median intensity of K310 glycated albumin peptides detected by mass spectrometry in the plasma of patients without (*n* = 29) and with DM (*n* = 39) (Welch’s *t* test). (**D**) Heatmap of 100 highest fold change differences between DM and non-DM platelet lysate proteomes in the resting state. 2,467 proteins were consistently detected in >50% of samples. Enrichment in proteins involved in response to oxidative stress (Z-scores shown). (**E**) Correlation of the top 100 upregulated platelet lysate proteins, in response to low-dose thrombin 0.025 U/mL, with serum fructosamine. SEC61B was the only platelet protein significantly correlated with serum fructosamine (red circle) (Spearman’s rho = 0.33, *P* = 0.029). ARMT1, CPNE2, and COMMD3 were negatively correlated with serum fructosamine (blue circles). (**F**) SEC61B, SEC61A, and SEC61G levels determined by mass spectrometry in platelets grouped in normal fructosamine (200–290 μmol/L) (*n* = 28) (gray) and high fructosamine (>290 μmol/L) (*n* = 15) (red) (Mann-Whitney test). Data are shown as the mean ± SD. GPX7, glutathione peroxidase 7; AMBP, α-1-microglobulin/bikunin precursor; MGMT, O-6-methylguanine-DNA methyltransferase; RWDD1, RWD domain containing 1; ARG1, arginase 1; SEPP1, selenoprotein P; HP, haptoglobin; APOA4, apolipoprotein A4; APOD, apolipoprotein D; APOE, apolipoprotein E; ARMT1, acidic residue methyltransferase 1; COMMD3, COMM domain containing 3; CPNE2, calcium-dependent phospholipid-binding protein; SEC61B, SEC61 translocon subunit β; SEC61A, SEC61 translocon subunit α; SEC61G, SEC61 translocon subunit γ.

The workflow of collection of clinical data and blood samples for the separation of plasma, serum, and washed platelets is shown in Figure 1A, and the clinical characteristics are shown in Table 1. As expected, we measured significantly higher serum fructosamine and plasma glycated albumin in patients with DM compared with those without DM (Figure 1, B and C). We utilized a low dose of thrombin (0.025 U/mL) to prime platelets for secretion, as described previously (14). Coronary artery disease severity scores, SYNTAX (17, 18), and Gensini (19) were not significantly different between patients with DM and patients without DM (Table 1). Platelet aggregation patterns of patients with DM showed a trend of increasing in response to low-dose thrombin, but there were no statistically significant differences between the DM and non-DM groups to standard dose agonists (Supplemental Data File 1 and Supplemental Figure 1; supplemental material available online with this article; https://doi.org/10.1172/JCI184597DS1).

### Oxidative stress and ER stress pathways are enriched in platelets in diabetes.

We isolated platelets from patients by serial centrifugation using our previously established method, which yielded high-purity platelets with low white cell (0.018% CD45^+^ events/total events) and red cell (0.013% CD235a^+^/total events) contamination (14). This was consistent with a platelet purity of 99.9% comparable with platelet isolation protocols used for proteomics and transcriptomics (20). Employing our established platelet proteomic platform (14), we identified 2,467 proteins across the samples. Proteins were ranked by fold change comparing DM and non-DM (Supplemental Data File 2, Resting platelet lysate proteome DM vs. non-DM). The top 100 highest fold change differences between DM and non-DM proteomes of resting platelet lysates were compared by Gene Ontology analysis. This revealed an enrichment for proteins involved in response to oxidative stress and cellular oxidant detoxification, such as glutathione peroxidase 7 (GPX7) and selenoprotein P (SEPP1) (Figure 1D), in line with oxidative stress being a feature of diabetes (2).

### Platelet SEC61B protein uniquely correlates with serum fructosamine.

We then correlated the top 100 lysate proteins, higher in fold change after thrombin stimulation in DM compared with non-DM groups, with their corresponding serum fructosamine abundance. We found that only SEC61B had a significant positive correlation with serum fructosamine (Figure 1E); which was independent of both scores used to assess coronary artery disease burden: SYNTAX (Spearman’s rho –0.011, *P* = 0.95) (17) and Gensini (Spearman’s rho –0.064, *P* = 0.69) (19).

SEC61B is the β subunit of the SEC61 translocon complex, which is a heterotrimeric complex including α, β, and γ subunits (9, 10). SEC61B (by mass spectrometry) was significantly elevated in platelets from patients with high fructosamine compared with patients with normal fructosamine, whereas the α (SEC61A) and γ (SEC61G) subunits of the translocon were not significantly different (Figure 1F).

**Figure 2:**
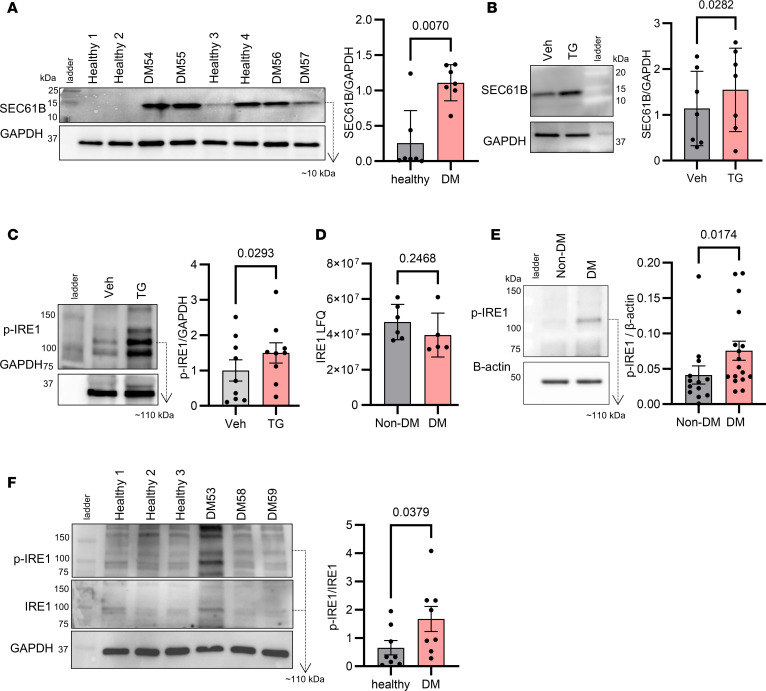
Platelets in type 2 diabetes have features of endoplasmic reticulum dysregulation evidenced by increased levels of SEC61B and activation of the ER stress sensor IRE1. (**A**) Representative Western blots of SEC61B in resting platelet lysate from healthy individuals and from patients with DM. Band intensity ratio of SEC61B to GAPDH in healthy (gray) versus DM platelets (red). *n* = 7 individuals per group (Mann-Whitney test). (**B**) Representative Western blots of SEC61B in platelets from healthy individuals treated with DMSO (vehicle) or thapsigargin (TG, 2 μM), an inducer of ER stress. Band density ratio of SEC61B to GAPDH of platelets treated with vehicle (gray) or TG (red). *n* = 7 individuals (paired *t* test). (**C**) Representative Western blots of p-IRE1 in platelets from healthy individuals treated with DMSO (vehicle) or TG (2 μM). Band density ratio of p-IRE1 to GAPDH of platelets treated with vehicle (gray) or TG (red). *n* = 9 individuals (paired *t* test). (**D**) IRE1 by mass spectrometry in platelets from patients with (*n* = 5) and without DM (*n* = 6) (Mann Whitney test). (**E**) Band intensity ratio of phosphorylated IRE1 (p-IRE1) to β actin of platelet lysates from patients without (*n* = 13) (gray) and with DM (*n* = 17) (red), as detected by Western blot (Mann-Whitney test). (**F**) Representative Western blots of p-IRE1, IRE1, and GAPDH in resting platelet lysate from healthy individuals and from patients with DM. Band intensity ratio of pIRE1 to IRE1 in healthy (gray) versus DM platelets (red). *n* = 8 individuals per group (Mann-Whitney test). Data are shown as the mean ± SD. Non-DM, no diabetes mellitus; DM, diabetes mellitus; LFQ, label-free quantification; p-IRE1, phosphorylated inositol requiring protein-1; IRE1, inositol requiring protein-1.

To validate our findings of increased platelet SEC61B in a separate cohort, we recruited 8 additional patients with type 2 DM and 8 individuals acting as healthy controls. Their clinical characteristics are shown in Table 2. We quantified the amount of SEC61B in their platelet lysates by Western blot, confirming that SEC61B was at low levels in healthy young individuals but was significantly increased in platelets from patients with type 2 DM (Figure 2A).

### Increased platelet SEC61B is associated with activation of the IRE1 ER stress sensor.

The SEC61 translocon has previously been shown to attenuate ER stress signaling through the IRE1 pathway (21), and recent evidence has identified activation of IRE1 in diabetic platelets (5). We sought to understand if SEC61B upregulation associates with IRE1 activation. By treating healthy platelets with TG (22), a known activator of IRE1, we found an increase in expression of SEC61B (Figure 2B) and phosphorylated IRE1 (p-IRE1), which is the active form of IRE1 generated upon ER stress (Figure 2C). Whereas the amount of total IRE1 (gene name ERN1) was not different between DM and non-DM platelets, as measured by mass spectrometry (Figure 2D), p-IRE1 was significantly increased in platelets in the DM group (by Western blot) (Figure 2E). We subsequently confirmed increased platelet p-IRE1, by Western blot, in our second cohort of patients compared with healthy individuals (Figure 2F). In contrast, ER protein glucose-regulated protein 78 (GRP78) (22) or phosphorylated eukaryotic initiation factor 2α (eIF2α) (downstream of the protein kinase R-like ER kinase [PERK] ER stress pathway) (22) was not increased in the high-fructosamine group (Supplemental Figure 2, A and B). These findings suggest that GRP78 and p-eIF2a are not markers of ER stress in our models.

### SEC61B is increased in conditions of hyperglycemia and ER stress.

**Figure 3:**
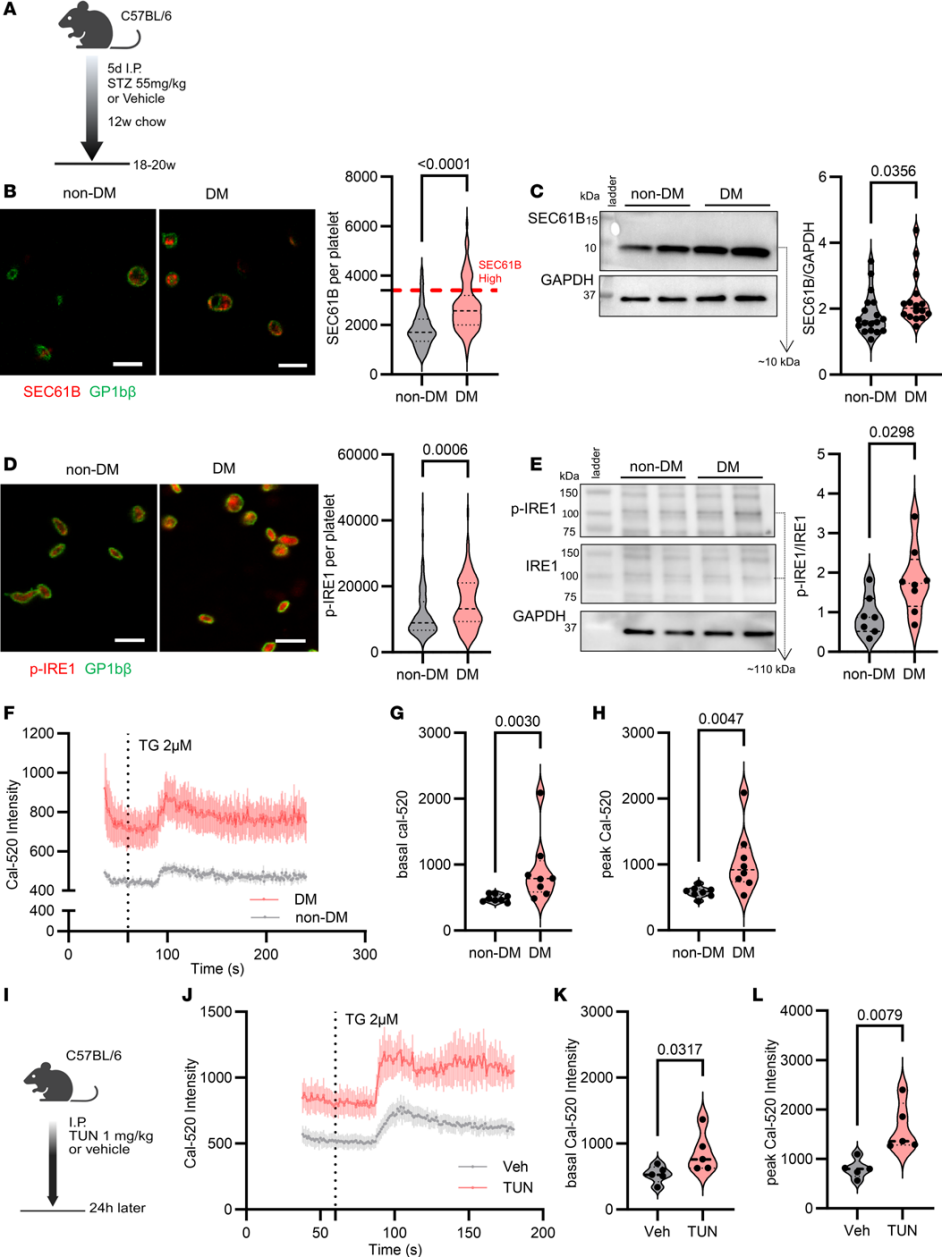
Hyperglycemia and tunicamycin (an ER stress inducer) increase platelet SEC61B expression and cytosolic calcium. (**A**) C57BL/6 mice were injected with streptozotocin (STZ) to induce diabetes (DM) or citrate buffer (vehicle, non-DM). (**B**) Representative images of platelets from non-DM and DM mice stained for SEC61B (red) and GP1Bb (green). SEC61B intensity of immunostained platelets from non-DM (gray) and DM (red) mice. Median (black dashed line), quartiles (black dotted line), and mean + 2SD (red dotted line) of SEC61B intensity in platelets from non-DM mice as cutoff for SEC61B “high” platelets are shown. (**C**) Representative Western blots of SEC61B in platelet lysates and SEC61B-to-GAPDH band intensity ratio in non-DM (gray) versus DM (red) platelet lysates. *n* = 16–18 mice per group. (**D**) Representative images of platelets from non-DM and DM mice stained for p-IRE1 (red) and GP1Bb (green). P-IRE1 intensity of immunostained platelets from non-DM (gray) and DM (red) mice. (**E**) Representative Western blots of p-IRE1, IRE1, and GAPDH and p-IRE1–to-IRE1 band intensity ratio in non-DM (gray) and DM (red) mice. *n* = 7–8 mice per group (Welch’s test). (**F**) Cytosolic calcium was quantified with Cal-520–loaded platelets from non-DM (gray) and DM (red) mice. SEC61-mediated ER calcium leak was elicited by TG treatment (solid line indicates the mean; shaded region indicates SEM). (**G**) Basal cytosolic calcium and (**H**) peak fluorescence intensity in platelets from non-DM (gray) and DM mice (red). *n* = 8 mice per group. (**I**) In vivo ER stress induction by tunicamycin (TUN, 1 mg/kg). (**J**) Cytosolic calcium measured in Cal-520–loaded platelets from DMSO-treated (vehicle, gray) or TUN-treated (red) mice before and after the addition of TG. (**K**) Basal cytosolic calcium and (**L**) peak calcium in platelets from vehicle-treated (gray) and TUN-treated mice (red). *n* = 5 per group. Mann-Whitney test was used for all comparisons unless otherwise specified. *n* = 15–20 platelets per mouse from, *n* = 3–5 mice per group in platelet immunofluorescence studies. Scale bars: 5 μm.

To further understand the effect of hyperglycemia on SEC61, we used the animal model of streptozotocin-induced (STZ-induced) hyperglycemia. C57BL/6J mice were injected with STZ to induce hyperglycemia (23, 24) (Figure 3A) and isolate their platelets. We found that platelets from hyperglycemic mice (DM) had increased platelet SEC61B by immunofluorescence compared with platelets from control mice (non-DM) (Figure 3B). Using the mean + 2SD of SEC61B intensity in platelets from non-DM mice as a cutoff, 24% of DM platelets were SEC61B “high” compared with 5% of non-DM platelets (Figure 3B). Increased SEC61B was confirmed by Western blot in resting DM platelets from a separate cohort of STZ-induced DM mice (Figure 3C). Resting platelets from DM mice also had evidence of ER stress, as they had increased p-IRE1, but not GRP78, compared with non-DM mice (Figure 3, D and E, and Supplemental Figure 2C), consistent with our findings in human DM platelets.

We sought to determine if increased platelet SEC61B in hyperglycemia is associated with increased calcium leak. Platelets isolated from hyperglycemic mice were loaded with calcium indicator dye Cal-520 (25), and cytosolic calcium was measured by flow cytometry over time (Figure 3F). Cal-520 fluorescence was significantly higher in DM platelets at baseline (Figure 3G) and in response to TG (Figure 3H). These findings suggest that increased platelet SEC61B in hyperglycemia is associated with increased ER calcium leak. Furthermore, DM platelets mobilize more calcium into the cytosol after activation with thrombin and U46619 in a dose-dependent manner, consistent with platelet hyperresponsiveness to agonists being a feature of diabetes (Supplemental Figure 3, A–D).

To answer the question of if increased ER calcium leak occurs downstream of activation of the IRE1 stress pathway, we utilized an in vivo model of activation of platelet IRE1 that we, and others, have recently described (5, 22). Mice were injected with tunicamycin 1 mg/kg i.p., which induces ER stress by inhibition of N-glycosylation, and platelets were collected after 24 hours (Figure 3I). Platelets from tunicamycin-treated mice had increased cytosolic calcium at baseline (Figure 3, J and K) and in response to ex vivo stimulation with TG (Figure 3L). These data support the hypothesis that increased platelet ER calcium leak can be elicited secondary to ER stress and is not limited to hyperglycemia.

### Increase of SEC61B in hyperglycemia originates at the megakaryocyte level.

**Figure 4:**
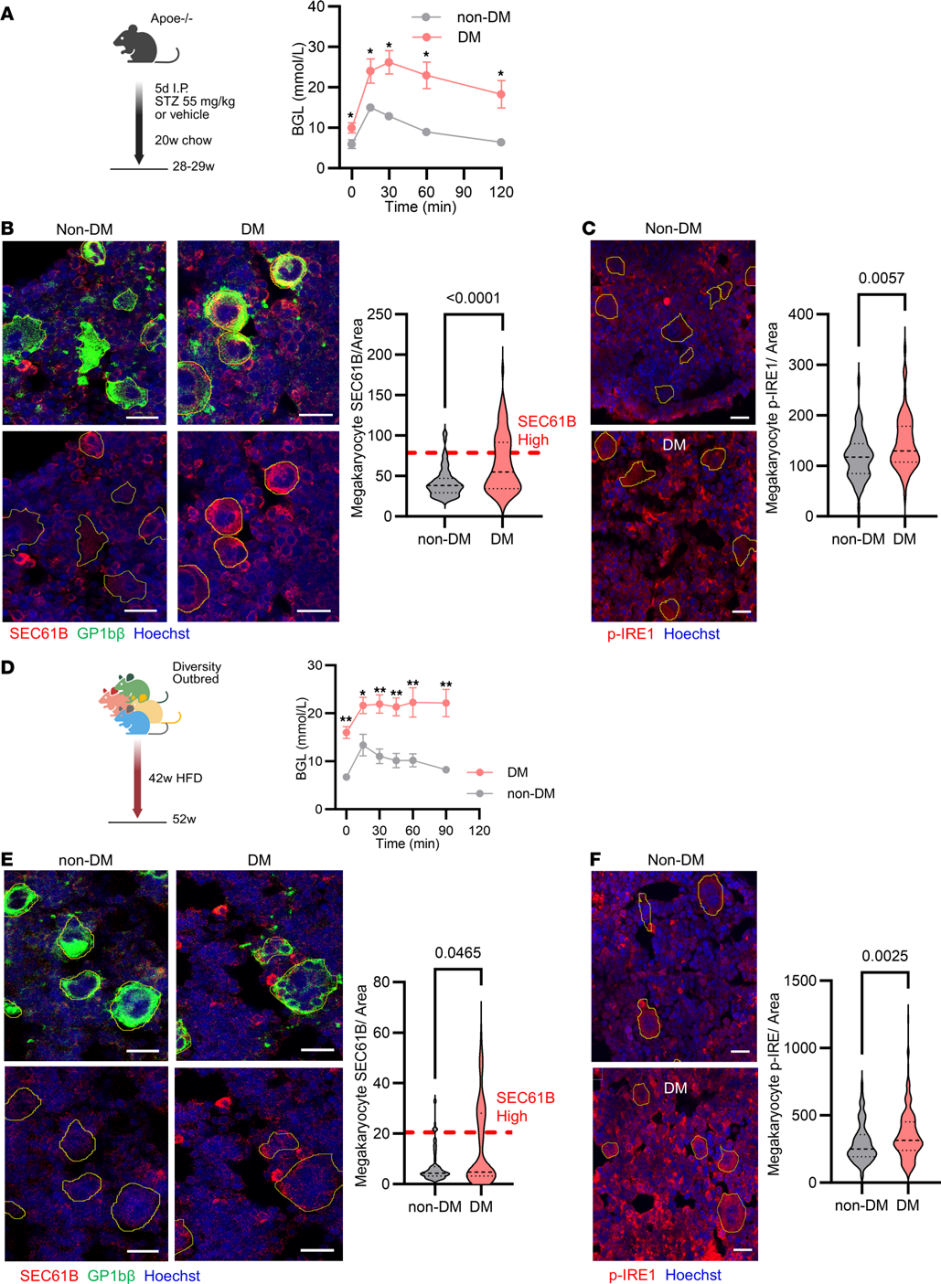
Increased SEC61B occurs at the level of the megakaryocyte in diabetic mice. (**A**) Apoe^–/–^ mice injected with STZ as a model of type 2 diabetes. Glucose tolerance test in vehicle-treated (non-DM) versus STZ-treated (DM) mice. (**B**) SEC61B fluorescence intensity in the bone marrow of non-DM (left) and DM (right) Apoe^–/–^ mice, stained for SEC61B (red) and GP1bβ (green). Nuclei are stained with Hoechst 33258 (blue). Representative images are shown. SEC61B fluorescence intensity per square pixel in megakaryocytes of non-DM or DM Apoe^–/–^ mice. Median (black dashed line), quartiles (black dotted line), and mean + 2SD of SEC61B intensity in vehicle-treated mouse megakaryocytes as cutoff for SEC61B “high” megakaryocytes are shown. 15–20 megakaryocytes /mouse were analyzed from *n* = 5 mice per group. (**C**) p-IRE1 in megakaryocytes (outlined) of non-DM (top) or DM (bottom) Apoe^–/–^ mice by immunofluorescence staining. p-IRE1 is shown in red, and nuclei are shown in blue. Representative images are shown. P-IRE1 fluorescence intensity per area of immunostained megakaryocytes of non-DM or DM Apoe^–/–^ mice. *n* = 15–20 megakaryocytes per mouse, *n* = 5 mice per group. (**D**) Diversity Outbred mice on high-fat diet as a model of type 2 diabetes. Glucose tolerance test in non-DM (normoglycemic) versus DM (hyperglycemic) mice. (**E**) SEC61B immunostaining of megakaryocytes in the bone marrow of non-DM (left) and DM (right) Diversity Outbred mice. (**F**) P-IRE1 immunostaining of megakaryocytes (outlined) of non-DM (top) or DM (bottom) Diversity Outbred mice. 15 –20 megakaryocytes per mouse were analyzed from *n* = 5 Diversity Outbred mice per group (Mann-Whitney test). For glucose tolerance test, multiple unpaired *t* tests with Benjamini, Krieger, and Yekutieli correction for multiple testing. *q < 0.05, **q < 0.01. STZ, streptozotocin; HFD, high-fat diet; GP1Bb, glycoprotein 1B β; p-IRE1, phosphorylated inositol-requiring enzyme 1. Scale bars: 20 μm.

To understand if upregulation of platelet SEC61B in hyperglycemia originates from megakaryocytes, we employed two additional mouse models of type 2 diabetes, available to us, for analysis of megakaryocyte SEC61B content. The first model was injection of STZ in Apoe^–/–^ mice (Figure 4A); this model has previously been used to model features of type 2 diabetes, including hyperlipidemia and hyperglycemia in platelet studies (24). The second was a Diversity Outbred mouse model (Diversity Outbred in Australia) fed on a high-fat diet (26). A major advantage of using Diversity Outbred mice is that, like humans, they are genetically and phenotypically heterogeneous with varying degrees of obesity and other metabolic defects characteristic of the metabolic syndrome in humans. Thus, as in humans, they can be grouped per phenotype (hyperglycemic vs. normoglycemic) rather than per genotype. The characteristics of the Apoe^–/–^ and outbred mice included in the study are shown in Table 3.

We found that SEC61B was increased in the megakaryocytes of DM versus non-DM Apoe^–/–^ mice (Figure 4B), and there was also an increase in the staining for p-IRE1 (Figure 4C) and p-eIF2α (Supplemental Figure 2D). Immunostaining of bone marrow from the Diversity Outbred mice (Figure 4D) showed increased SEC61B in DM mice compared with non-DM mice (Figure 4E) and an increase in the staining for p-IRE1(Figure 4F) but not p-eIF2α (Supplemental Figure 2E). Using the mean + 2SD of SEC61B intensity in megakaryocytes from normoglycemic mice as a cutoff, approximately 35% of megakaryocytes from hyperglycemic mice were SEC61B “high” (Figure 4, B and E). ER chaperone GRP78 was unchanged in megakaryocytes of DM Apoe^–/–^ and DM Diversity Outbred mice (Supplemental Figure 2, F and G), compared with their respective non-DM controls.

### Increased SEC61B expression in HEK293 cells inhibits protein synthesis without activation of IRE1.

**Figure 5:**
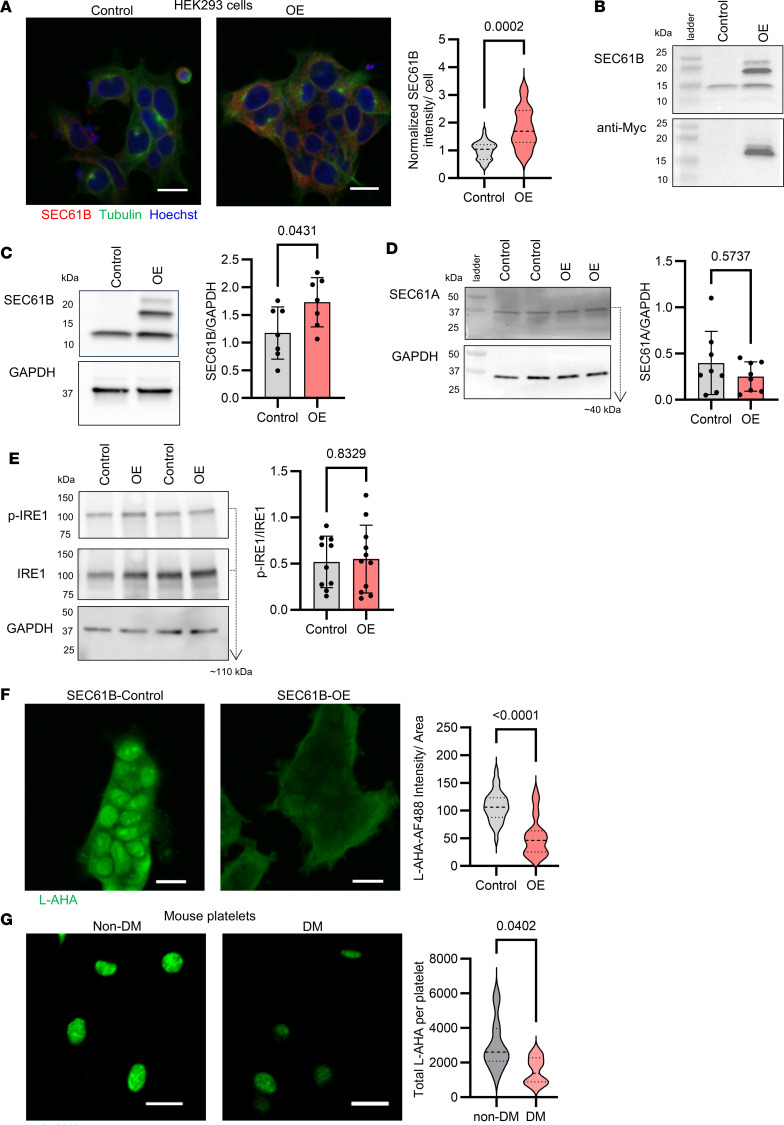
SEC61B overexpression in HEK cells does not induce activation of IRE1 but is associated with decreased protein synthesis. (**A**) SEC61B (red) and tubulin (green) immunostaining of lentiviral vector-transfected control or SEC61B-OE HEK293 cells. Nuclei are stained with Hoechst 33258 (blue). Representative images are shown. Scale bar: 20 μm. Normalized SEC1B intensity/cell in control (gray) and OE (red) cells. *n* = 20–30 cell clusters from *n* = 3 independent experiments (Welch’s test). (**B**) Representative Western blots of SEC61B, anti-Myc, and GAPDH of control and OE cells. SEC61B in HEK293 OE cells runs as 2 bands: native SEC61B (~10 kDa) and Myc-tagged SEC61B (~15 kDa). (**C**) Band intensity ratio of SEC61B to GAPDH in lysate of control (gray) versus OE cells (red). *n* = 7 independent experiments per group (Welch’s test). (**D**) Representative Western blots of SEC61A and GAPDH of control and OE cells. Band intensity ratio of SEC61A to GAPDH in lysate of control (gray) versus OE cells (red). *n* = 8 independent experiments (Welch’s test). (**E**) Representative Western blots of p-IRE1, IRE1, and GAPDH of control and OE cells. Band intensity ratio of p-IRE1 to IRE1 in HEK293 lysate of control (gray) versus OE cells (red). *n* = 10 independent experiments (Welch’s test). (**F**) Representative images of L-AHA fluorescence intensity (green) as a measure of protein synthesis in control and SEC61B-OE cells. Scale bar: 20 μm. L-AHA fluorescence intensity per cell area measured in *n* = 30 cell clusters per group from *n* = 3 independent experiments (Mann Whitney test). (**G**) Representative images of L-AHA fluorescence intensity (green) in platelets from normoglycemic (non-DM) and streptozotocin-induced hyperglycemic (DM) mice. Scale bar: 5 μm. L-AHA fluorescence intensity per platelet in non-DM (gray) and DM (red) mice. 20 platelets analyzed per mice, *n* = 7–8 mice per group (Welch’s *t* test). SEC61B OE, SEC61B overexpressing cells; L-AHA, L-azidohomoalanine.

To model the effects of increased SEC61B in a cell system, we overexpressed the β subunit of SEC61 in HEK293 cells. Transfection of HEK293 cells with a lentiviral *SEC61B*-overexpression (*SEC61B*-OE) vector induced an increase in SEC61B expression measured by immunofluorescence, compared with that in cells transfected with a control vector by immunofluorescence (Figure 5A). Western blot confirmed successful transfection with detection of the c-myc–tagged transfected SEC61B in OE cells (Figure 5B) and a 1.7-fold increase in SEC61B in OE cells compared with control cells (Figure 5C). Overexpression of SEC61B did not alter the expression of SEC61A in OE cells (Figure 5D) and was not accompanied by the activation of IRE1 (Figure 5E) or eIF2α (Supplemental Figure 4, A and B). These findings support that overexpression of SEC61B by itself does not induce ER stress in our HEK293 model.

In contrast, overexpression of SEC61B led to decreased protein synthesis in HEK293 cells consistent with the described decrease in protein transport by SEC61 in the setting of ER calcium loss (27). De novo protein synthesis was determined by incorporation of L-azidohomoalanine (L-AHA) as previously described (28). *SEC61B*-OE cells showed a significant decrease in protein synthesis by 51% compared with controls (Figure 5F). Similarly, platelets from DM C57BL/6 mice had decreased protein synthesis as measured by L-AHA incorporation ex vivo, compared with platelets from non-DM mice (Figure 5G).

### Increased SEC61B expression enhances ER calcium leak.

**Figure 6:**
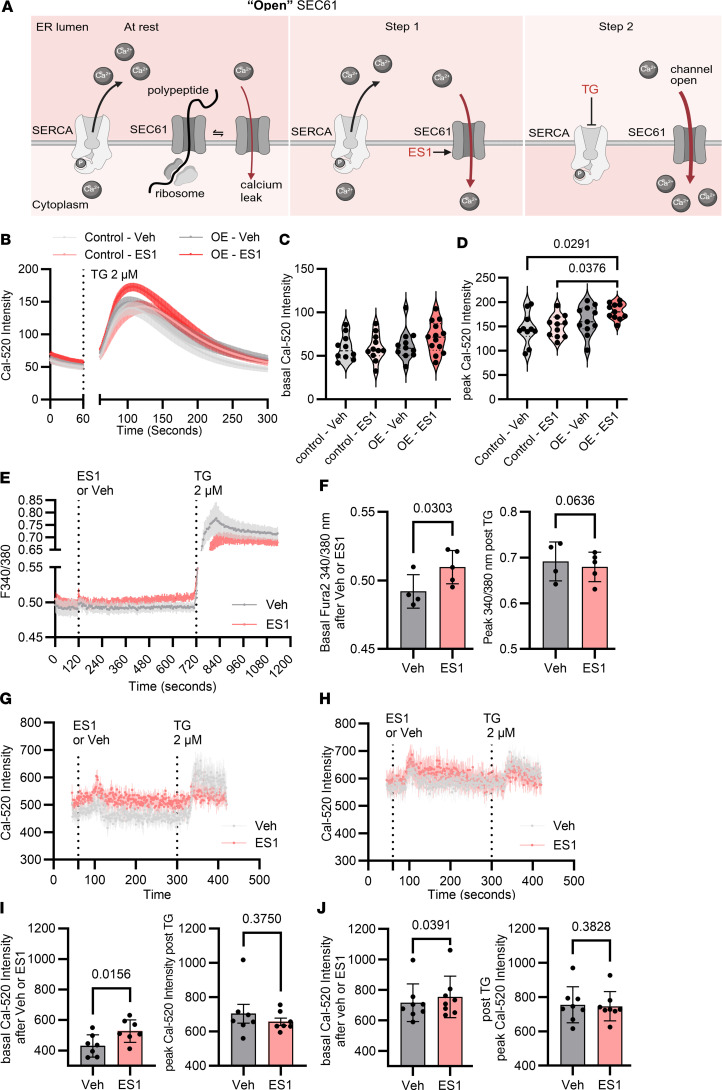
Eeyarestatin I, which stabilizes SEC61 in its “open” — calcium permeable — conformation, increases ER calcium leak in HEK293 cells overexpressing SEC61B and in platelets. (**A**) Schematic for 2-step induction of SEC6 calcium leak. In step 1, eeyarestatin I (ES1) “opens” the SEC61 channel allowing calcium to leak from the ER into the cytosol. In step 2, TG inhibits SERCA from pumping calcium back into the ER to measure maximal SEC61-mediated ER calcium leak. (**B**) Cytosolic calcium was quantified over time in Cal-520–loaded control or SEC61B-overexpressing (SEC61B-OE) HEK293 cells using a fluorescent plate reader. HEK293 control and OE cells were treated with vehicle (gray) or ES1 (red) for 1 minute, followed by addition of TG, in the presence of EGTA (solid line indicates mean; shaded region indicates SEM). (**C**) Basal and (**D**) peak fluorescence intensity of Cal-520 in the presence of vehicle or ES1 in control (gray) and OE cells (red). *n* = 10 independent experiments (1-way ANOVA with Dunn’s multiple comparisons test). (**E**) Cytosolic calcium was quantified over time in Fura2-loaded human platelets in the presence of vehicle or ES1 50 μM for 10 minutes, followed by addition of TG. (**F**) Basal and peak fluorescence intensity of Fura2 10 minutes after incubation with vehicle or ES1 (basal) and after addition of TG (peak). Mean ± SD, *n* = 5 healthy donors (Mann-Whitney test). (**G** and **H**) Cytosolic calcium was quantified over time in Cal-520–loaded platelets from (**G**) normoglycemic (non-DM) mice and (**H**) streptozotocin-induced hyperglycemic (DM) mice. Platelets were treated with ES1 for 5 minutes, followed by TG, in the presence of EGTA (solid line indicates mean; shaded region indicates SEM). (**I** and **J**) Basal and peak fluorescence intensity in Cal-520–loaded platelets from vehicle-treated (Veh, gray) versus ES1-treated (red) platelets from (**I**) non-DM mice and (**J**) DM mice. *n* = 7 mice, per group (Welch’s *t* test). SERCA 2b, sarco/endoplasmic reticulum calcium ATPase; ES1, eeyarestatin I; TG, thapsigargin.

We employed chemical modulators of SEC61 and SERCA (Figure 6A) to interrogate the role of the SEC61 translocon in platelet ER calcium leak. All current modulators of the SEC61 translocon act on the SEC61A subunit (27, 29, 30), with no SEC61B-specific inhibitors available to our knowledge. Eeyarestatin (ES1) promotes the SEC61 channel to adopt an “open,” calcium-permeable, state (31). ES1 is predicted by docking studies to bind to the lateral gate of SEC61 α subunit that facilitates insertion of transmembrane segments into the lipid bilayer (32, 33). Exposure to ES1 is associated with ER stress; this is time dependent and has only been reported to occur following hours of treatment (34, 35). In contrast, HEK293 cells and platelets were exposed to ES1 for only minutes during the calcium flux assays in this study, with protein translation and translocation not thought to be affected in this time frame (35). TG inhibits SERCA preventing the reentry of calcium into the ER (33). The net effect of “opening” the SEC61 translocon and inhibiting SERCA was to induce maximal SEC61-mediated ER calcium leak (Figure 6A). Inducing ER calcium leak in HEK293 cells by treatment with ES1, followed by TG, resulted in significantly increased cytosolic calcium in *SEC61B*-OE cells compared with controls by 38.8% (Figure 6, B–D).

In separate experiments we knocked down *SEC61B* in HEK293 cells using CRISPR/Cas 9. Two CRISPR guides (designated g1 and g2) were used to transfect HEK293 cells, resulting in 2 knockout lines (KO-1 and KO-2). After puromycin clone selection, SEC61B expression was reduced by 41.5%–77.7% in the SEC61B-KO cell lines (Supplemental Figure 5, A–C), whereas the levels of SEC61A were unaffected (Supplemental Figure 5D). ER calcium efflux was significantly increased with *SEC61B* depletion (Supplemental Figure 5, E and F).

The KO cells had a significantly lower rate of de novo protein synthesis, as determined compared with the control cells (mean 57.7% reduction for KO-1 and 71.9% reduction for KO-2 compared with KO control, Supplemental Figure 5G).

Our data with both *SEC61B*-OE and -KO HEK293 cells support that disrupting the stoichiometry of SEC61 complex may affect the function of SEC61 translocon.

SEC61 translocon subunits have been reported in platelet transcriptomic and proteomic studies (36, 37), but the function of the translocon in platelets has not been described. In healthy human donor platelets, we observed slow calcium leak after exposure to ES1 with a subsequent decrease in translocon-mediated peak calcium flux (Figure 6, E and F).

To probe the role of SEC61-induced ER calcium leak by ES1 in diabetes, calcium flux was studied in platelets from STZ-injected (DM) mice and non-DM control mice. Treatment of platelets with ES1 led to an increase in basal cytosolic calcium in both non-DM and DM platelets (Figure 6, G–J). However, diabetic platelets had higher basal cytosolic calcium before the addition of ES1, and a relatively blunted increase after ES1, suggesting that baseline “leakiness” of the translocon may have already reached a plateau in hyperglycemic platelets.

### Inhibition of SEC61 with ANX decreases cytosolic calcium and platelet aggregation in vitro and in vivo.

**Figure 7:**
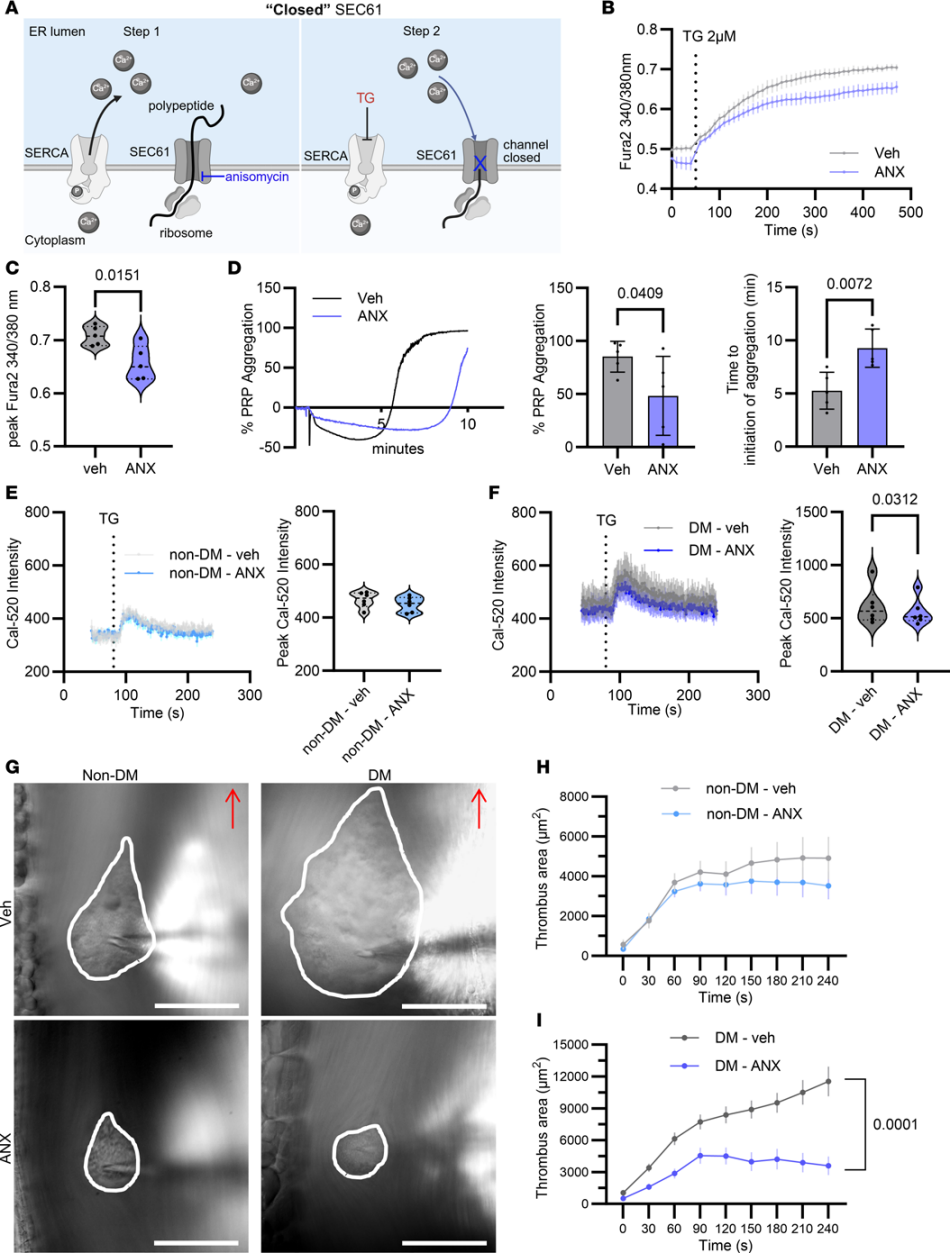
Anisomycin, which promotes a “sealed” SEC61 conformation, decreases ER calcium leak in HEK293 cells and platelets and inhibits platelet thrombus formation in vivo. (A) Schematic of inhibition of SEC61 by anisomycin (ANX). ANX inhibits ribosomal elongation of the peptide, locking the peptide within the pore, thus preventing calcium leak when SERCA2 is inhibited with TG. **(B)** Cytosolic calcium was quantified with Fura2-loaded human platelets in response to TG, after pretreatment with vehicle or ANX (200 μM for 2 hours). Solid line indicates mean; shaded region indicates SEM. **(C)** Peak Fura2 in platelets treated with vehicle (gray) or ANX (blue) after TG addition. *n* = 6 healthy donors (Welch’s *t* test). **(D)** Platelet aggregation over time in response to TG, after pretreatment of platelet-rich plasma (PRP) with vehicle or ANX 200 μM for 2 hours. Maximal percentage aggregation of PRP, and time to initiation of platelet aggregation, in response to TG, after pretreatment with vehicle (gray) or ANX (blue). *n* = 5 healthy donors (Welch’s *t* test). **(E)** Cytosolic calcium in Cal520-loaded non-DM platelets after treatment with vehicle (gray) or ANX (blue) (100 μM for 1 hour), prior to TG addition. Peak Cal520 in non-DM platelets treated with vehicle (gray) or ANX (blue) followed by TG. *n* = 6 mice per group (Welch’s t test). **(F)** Cytosolic calcium in Cal520-loaded DM platelets, as described before. *n* = 6 mice per group (Welch’s t test). **(G)** Differential interference contrast images depicting thrombi (dotted line) in mouse mesenteric venules forming 4 minutes after needle insertion in non-DM and DM mice. Mice were pretreated with i.v. vehicle control (Veh) or ANX (20 mg/kg, 1.5 hours prior to surgery). The scale bar is 50 μm.**(H)** Surface area of thrombi generated in non-DM and **(I)** in DM mice was quantified at the indicated time points after needle insertion. Results are expressed as mean ± SD. *n* = 3–5 mice per group, 6–8 thrombi per mouse (2-way ANOVA).

The SEC61 translocon modulator ANX inhibits the ribosome, resulting in translational arrest and the trapping of the nascent polypeptide within the channel. This causes a “sealed” translocon, preventing calcium leakage in response to TG (12, 38) (Figure 7A). Using doses between 10 and 400 μM of ANX in HEK293 cells, we found that the dose of 200 μM for 2 hours inhibited calcium flux (Supplemental Figure 4, C and D). Incubation of healthy human platelets with ANX (200 μM for 2 hours) led to a decrease in calcium flux in response to TG 2 μM (Figure 7, B and C). Furthermore, ANX-treated human platelets showed decreased aggregation and a delay in the initiation of aggregation in response to TG (2 μM) (Figure 7D), whereas incubation of healthy blood with ANX decreased platelet adhesion to a fibrinogen-coated microfluidic channel under shear (1,000/s) (Supplemental Figure 4, E–G, and Supplemental Video 1, Anisomycin and platelet adhesion).

To study the role of ANX in calcium flux in diabetes, we treated platelets isolated from non-DM and DM mice with 100 μM ANX for 1 hour and measured cytosolic calcium, after stimulation with TG, by using Cal-520 florescence. Non-DM platelets, treated with ANX, showed a nonsignificant decrease in cytosolic calcium after stimulation with TG (Figure 7E), whereas DM platelets, treated with ANX, had a significant decrease in cytosolic calcium in response to TG (Figure 7F).

We further investigated the in vivo relevance of ANX treatment in the thrombotic tendency of DM mice. STZ-injected DM and vehicle-injected non-DM mice received 20 μg/g ANX by tail vein injection (39). After 2 hours, needle in situ injury of mesenteric venules was performed, and platelet thrombus formation was recorded over time. The needle in situ model is an established model of hyperglycemia-dependent platelet thrombosis (23). We found that ANX-treated DM mice had significant inhibition of platelet thrombus formation compared with vehicle-treated mice. In contrast, non-DM mice developed smaller thrombi compared with DM mice with vehicle control, as expected (23), but thrombus size was not affected by ANX (Figure 7, G–I, and Supplemental Video 2, Anisomycin and thrombus formation). The inhibitory effect of ANX suggests that SEC61 inhibitors may have potential to reduce platelet hyperreactivity in diabetes.

### Increased ER calcium leak is associated with disturbed platelet ER homeostasis.

We sought to investigate whether the degree of ER calcium loss affects platelet secretion. For this we used two separate SERCA inhibitors: SERCA2 inhibitor (TG) and SERCA3 inhibitor (2,5-di-(tert-butyl)-1,4-benzohydroquinone [BHQ]) (8, 22, 40).

TG (“potent” SERCA inhibitor) produced a greater increase in cytosolic calcium in human platelets compared with BHQ (“moderate” SERCA inhibitor) (Supplemental Figure 6A). TG produced greater mobilization of α granule content, as evidenced by increased platelet surface CD62P (Supplemental Figure 6B) and greater activation of αIIbβ3, as determined by the PAC-1 binding (Supplemental Figure 6C).

Whereas TG activates the IRE1 ER stress pathway, BHQ does not induce p-IRE1 or SEC61B. Furthermore, neither TG nor BHQ induced p-eIF2α (Supplemental Figure 6, D and E). The more potent TG (but not BHQ) was able to mobilize ER proteins, including protein disulfide isomerase (PDI) and ER protein 5 (ERp5) to the platelet surface (Supplemental Figure 6, F and G), but this was not associated with the concurrent release of the same proteins (Supplemental Figure 6, H and I). The differential response to TG and BHQ supports that increasing platelet ER calcium loss is associated with mobilization of ER proteins to the platelet surface, which may further promote platelet hyperreactivity (22).

### Platelets from patients with diabetes have increased protein secretion in response to low-dose thrombin stimulation.

**Figure 8:**
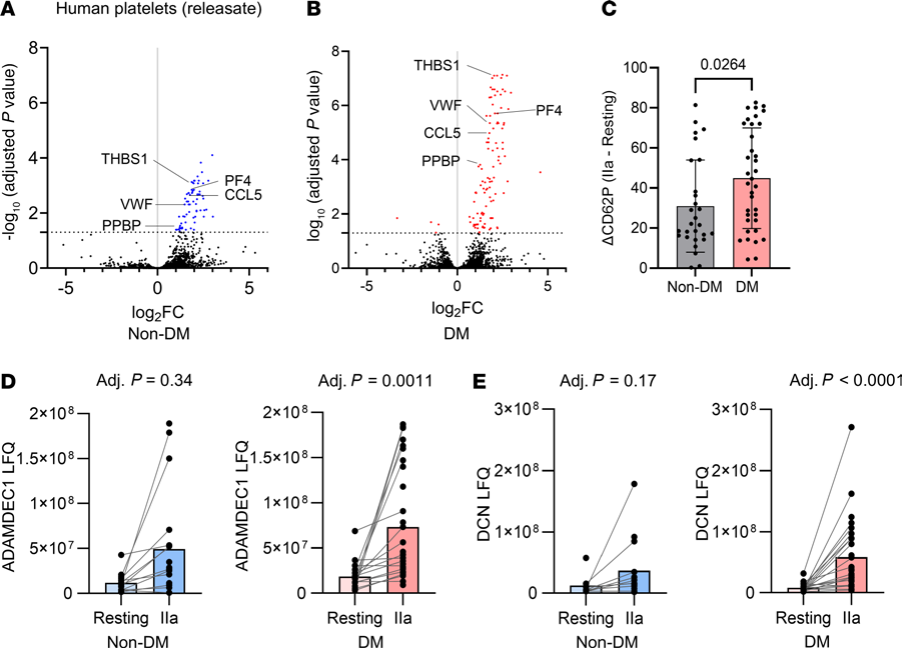
Platelets in diabetes secrete a broad range of proteins in response to submaximal stimulation that are involved in inflammation and atherosclerosis. (**A**) Volcano plots of proteins detected in the releasate of nondiabetic (non-DM) and (**B**) diabetic (DM) platelets after stimulation with low-dose thrombin (IIa) 0.025 U/mL. Released proteins that were significantly increased after stimulation with low-dose thrombin compared with released proteins in resting platelets are shown in blue for non-DM platelets and I red for DM platelets. (**C**) Difference of platelet surface CD62P expression before and after low dose IIa from patients without and with diabetes (Mann-Whitney test). (**D**) Secretion of ADAM like decysin 1 (ADAMDEC1) and (**E**) decorin (DCN) into the releasate of non-DM (blue) and DM (red) platelets following stimulation with low-dose IIα (Limma moderated *t* test). THBS1, thrombospondin I; VWF, von Willebrand factor; PPBP, proplatelet basic protein; PF4, platelet factor 4; CCL5, chemokine (C-C motif) ligand 5 (RANTES).

We treated platelets isolated from patients with or without DM with low-dose thrombin (0.025 U/mL) to prime the hyperreactivity of DM platelets to low-dose stimulus. We had previously determined this dose, by titration studies in healthy human platelets, to be required for submaximal (40%) aggregation of washed platelets (14). After low-dose thrombin stimulation a total of 109 and 71 proteins were significantly secreted by DM and non-DM platelets, respectively (Figure 8, A and B; Supplemental Table 2; Supplemental Data File 3, DM platelet releasate proteome; and Supplemental Data File 4, non-DM platelet releasate proteome). The released proteins shared similarities with previously described platelet protein releasates from patients with coronary artery disease in response to high-dose thrombin (1 U/mL) (41). We found that low-dose thrombin stimulation mobilized more α-granule CD62P in platelets in the DM group compared with the non-DM group (Figure 8C). Forty-six proteins were significantly released only by DM platelets. These included ADAM like decysin 1 (ADAMDEC1) (Figure 8D), a soluble protease that cleaves platelet-secreted proepidermal growth factor (pro-EGF) to high-molecular-weight EGF (42), which contributes to increased thrombosis in an in vivo carotid injury model (43), and decorin (Figure 8E), which interacts with integrin α_2_β_1_ on platelets, leading to platelet activation (44). ADAMDEC1 has previously been shown within the platelet proteome localized to the granule and membrane fractions (42), whereas decorin is a proteoglycan and, therefore, may be located to the plasma membrane but has not been described in platelets before (Supplemental Table 2).

## Discussion

Increased platelet reactivity remains a critical issue in diabetes, as patients derive less benefit from antiplatelet therapy and have elevated cardiovascular risk (1). Identifying alternative mechanisms of platelet hyperreactivity may offer new therapeutic options. We previously described a high-sensitivity proteomic platform that identified a novel platelet O-fucosylation (14, 45) and applied this methodology to identify altered protein pathways in diabetes. Prior platelet proteomic studies in diabetes have been limited to small cohorts or older methods of in-gel analysis (46, 47). Our study provides the largest proteomic analysis of intracellular and released platelet proteins in diabetes with coronary artery disease to date. The proteomic dataset is publicly available at the Proteomics Identifications Database (PRIDE; https://www.ebi.ac.uk/pride/archive/projects/PXD049321).

Our most important finding was increased platelet SEC61B in hyperglycemia. While present in healthy human and Apoe^–/–^ mouse platelets (37, 48), the functional role of SEC61B has not been explored. SEC61B upregulation seems to be a bone fide hyperglycemia phenomenon, as we confirmed SEC61B upregulation in a separate human diabetic cohort and in STZ-induced hyperglycemic mice. Functionally, increased SEC61B was associated with increased cytosolic calcium, while SEC61 inhibition decreased calcium leak and platelet aggregation, implicating SEC61B in platelet hyperreactivity in diabetes.

Platelets from people with DM show elevated basal cytosolic calcium (3, 4), partly due to impaired SERCA2 from oxidative stress (12). Although we did not observe increased STIM1 protein, its mRNA elevation in other studies (49) may reflect compensatory responses to calcium leak. Given SEC61’s role as a calcium leak channel in nucleated cells, we propose that SEC61B contributes to calcium dysregulation in diabetes.

SEC61B upregulation likely occurs secondary to platelet ER stress in hyperglycemia, as SEC61B was also upregulated following chemical induction of ER stress. The *SEC61B* mRNA has been shown to closely associate with the ER, which may explain its rapid protein synthesis after platelet ER stress (50). The spliced form of XPB1, the transcription factor downstream of IRE1 activation, binds the *SEC61B* promoter in chromosomal immunoprecipitation assays (51). These observations support the potential for *SEC61B* induction both in the megakaryocytes, via transcriptional and translational upregulation, and also via translational upregulation in the anucleate platelet.

Increased expression of SEC61B provides a positive feedback loop to further increase platelet ER stress due to the loss of ER calcium (52), a known trigger of ER stress (53). The SEC61 translocon is permeable to calcium when the pore “breathes” after releasing a polypeptide but appears calcium impermeable in the closed state (54, 55). How the β subunit of SEC61 regulates calcium leak through the SEC61 channel is intriguing, as it is peripheral to the pore of the channel. Calcium movement through the translocon occurs via the SEC61A pore (31); however, SEC61B and SEC61G have been proposed to play regulatory roles (56). Although there is no information on the role of SEC61B in calcium flux, correct stoichiometry of channel subunits may be necessary for optimal function of the channel. Unlike SEC61A/G, SEC61B lacks bacterial orthologs (57, 58), suggesting a eukaryote-specific role in protein synthesis and microtubule interaction (59, 60). Overexpression may destabilize translocon assembly, affecting peptide transfer and calcium flux (61, 62).

Another possible explanation for the increased calcium leak in SEC61B overexpression may be from decreased peptide transfer (11). Decreased peptide transfer may provide the opportunity for calcium to leak through the SEC61 channel into the cytosol. *SEC61B* overexpression or KO reduced protein synthesis, as evidenced by decreased L-AHA incorporation, further supporting the importance of the β subunit in optimal SEC61 function.

Apart from SEC61B, our proteomic analysis of intracellular proteins highlighted the enrichment of oxidation pathways in diabetic platelets. Diabetic platelets had increased levels of redox proteins GPX7 and SEPP1, which modulate H_2_O_2_ and ER oxidative folding (63, 64, 65). Redox imbalance activates platelets via AGE-CD36 interaction, with CD36 deficiency shown to be protective in thrombosis (66). Other danger signals (oxLDL, S100A9) act via CD36 (67), whereas ERK5 phosphorylation also enhances platelet activation (68).

Our analysis of platelet releasate proteins revealed that diabetic platelets secreted a wider range of proteins compared with nondiabetic platelets, including inflammation-associated proteins decorin and ADAMDEC10 (42, 43). These proteins, along with modular calcium-binding protein 1 (SMOC1), which promotes platelet responsiveness to thrombin (69), PDGF, which promotes the calcification of vascular smooth muscle cells in atherosclerosis (70), and neutrophil-derived S100A8/A9, create a proinflammatory, proatherosclerotic milieu (14, 24, 69, 70, 71).

We acknowledge limitations in human sample variability, including the heterogeneity among participants and interindividual variability in platelet responses to stimuli, which introduce imprecision. To increase the precision, we stringently adhered to the method of platelet isolation and measurements and validated our findings of SEC61B upregulation in a separate cohort of patients. We have tried to eliminate confounders by matching patients in DM and non-DM groups by age, sex, lipid levels, smoking status, and medications (72). The possibility of other confounders, such as hypertension, cannot be excluded. The use of the STZ mouse model as a “hyperglycemia-only” model in all platelet studies limits these confounders.

In summary, we have identified that SEC61 translocon plays a functional role in platelet biology as a calcium leak channel. SEC61B is a hyperglycemia-responsive regulator of calcium flux in platelets. Its upregulation in diabetes may contribute to platelet hyperreactivity and has potential as a biomarker or therapeutic target in cardiovascular disease.

## Methods

### Sex as a biological variable.

We included both male and female sexes in human and mouse studies. Sex was not considered as a biological variable in the studies.

### Supplemental materials.

Details regarding human platelet aggregation, platelet proteomic studies, platelet and megakaryocyte immunofluorescence, in vitro studies of platelet ER stress, Western blot, flow cytometry, generation of SEC61B KOs and overexpressors, HEK293 calcium flux assays, needle in situ model, and intravital microscopy are provided in the Supplemental Methods.

### Patient cohort.

Clinical data, including age, sex, body mass index, systolic and diastolic blood pressure, antiplatelet use, and glycated hemoglobin (HbA1c), were derived from electronic medical records. Serum cholesterol, triglycerides, HDL, and LDL were analyzed in a routine chemical pathology lab. As HbA1c measurements were not available for all patients, serum fructosamine and glycated albumin were measured as markers of glycemic control (15, 73). Serum fructosamine was measured by spectrophotometry in a routine chemical pathology lab. Patient fructosamine was classified as high (>290 μmol/L, approximately equivalent to HbA1c >7.0%) (15) and normal (<290 μmol/L, equivalent to HbA1c <7.0%) (normal range 200–290 μmol/L). Glycated albumin in plasma was measured by mass spectrometry for all patients (74). Coronary angiograms were reviewed and scored for Gensini and SYNTAX (17–19) (as surrogate markers for coronary disease burden) by two interventional cardiologists, and disagreement was resolved by consensus.

### Mouse models of diabetes.

Apoe^–/–^ mice were from The Jackson Laboratory.

Diversity Outbred mice from Australia (DOz) mice were established and sourced in-house.

C57BL/6J mouse were from Australian Bio Resources. Apoe-KO (Apoe^–/–^) mice, treated with STZ, were used as a model of type 2 DM, whereas Apoe^–/–^ mice, treated with citrate (vehicle), were used as controls (non-DM) (24). DOz mice were grouped into mice with hyperglycemia (DM) and mice with normoglycemia (non-DM) (26, 75). C57BL/6J mice (Australian Bio Resources) treated with STZ (23) were used as models of hyperglycemia (DM), whereas mice treated with citrate were used as control (non-DM).

Eight- to 9-week-old male and female Apoe^–/–^ mice were treated with either citrate vehicle or STZ 75 mg/kg over 5 consecutive days via i.p. injection (STZ, *n* = 3 male and *n* = 2 female; vehicle, *n* = 2 male and *n* = 3 female). As female mice are relatively more resistant to STZ compared with male mice, female mice received a second course of STZ 11 weeks after the first treatment course. Mice were fed a chow diet for 20 weeks before their study.

DOz mice were given ad libitum access to a high-fat diet made in-house containing 45% fat, 35% carbohydrate, and 20% protein from 10 weeks of age for 42 weeks. The characteristics and diet of these mice have been previously described (26, 75).

Six- to 8-week-old male C57BL/6J mice were treated with STZ 55 mg/kg i.p. daily for 5 consecutive days (23). Blood glucose levels were measured 1 week after STZ injection and monitored weekly during their maintenance and prior to experiments. Mice were fed with a chow diet and deemed hyperglycemic when the random blood glucose was confirmed to be greater than 15 mmol/L. Hyperglycemic mice were used 12 weeks after STZ injection.

Blood glucose was measured by glucometer (LifeSmart). All Apoe^–/–^ STZ mice and DOz mice had a glucose tolerance test performed at 14 weeks (Figure 4, A and D). DOz mice had a glucose measurement at 40 weeks and prior to their study.

### Mouse platelet calcium flux assay.

Diluted mouse platelets were obtained by 2 rounds of serial centrifugation (240*g*, 2 minutes, soft brake) of mouse whole blood (1:15, whole blood, buffer) with HEPES-Tyrode’s buffer (136.5 mM NaCl, 2.68 mM KCl, 20 mM NaHCO_3_, 1.5 mM Na_2_HPO_4_, 20 mM HEPES, 5.55 mM glucose) with bovine serum albumin (BSA, 0.35% w/v) containing Cal-520–AM (2 μM, Abcam) and probenecid (2 mM, Sigma Aldrich). Separately, the same experiments with Cal-520 were done in the absence of probenecid.

Diluted mouse platelets were incubated at 37^o^C in the dark for 30 minutes. The platelets were then diluted in an equal volume of HEPES-Tyrode’s buffer with BSA and incubated at room temperature in the dark for 15 minutes to allow Cal-520–AM deesterification. The diluted platelets were incubated with calcium chloride (1 mM) for at least 3 minutes to allow the platelets to recalcify. Subsequently, EGTA (1 mM) was added to chelate residual extracellular calcium.

### Mouse platelet calcium flux in response to SEC61 modulators.

To assess platelet calcium response to ES1 (Cayman Chemicals), an ER-associated degradation inhibitor that enhances SEC61-mediated calcium leak (32), ES1 diluted in HEPES-buffered Tyrode’s buffer (50 μM final concentration) or equal volume of DMSO vehicle was added manually to diluted platelets after the baseline recording. The ER calcium leak induced by ES1 was observed by recording for 180 seconds. Then, ER calcium leak was subsequently elicited by addition of SERCA inhibitor TG (2 μM, Merck Life Science) with events recorded for a further 120 seconds. Platelet cytosolic calcium levels were recorded using a BD Accuri 6 flow cytometer at the “slow” speed (14 μL/min), and a baseline of 60 seconds was recorded for all experiments.

To assess platelet calcium response to ANX, a ribosome inhibitor that prevents SEC61-mediated ER calcium leak (16, 38), diluted murine platelets were preincubated with 100 μM ANX or equal volume of DMSO vehicle control for 30 minutes, prior to loading with Cal-520 AM, as above. After the baseline recording, TG (2 μM) or vehicle (equal volume DMSO) was added, and events recorded for a further 180 seconds.

### Mouse platelet calcium flux in response to platelet agonists.

In agonist-dose curves, mouse platelet-rich plasma (PRP) was prepared by diluting whole blood (1:1) in HEPES-buffered Tyrode’s buffer followed by centrifugation at 240*g*, 2 minutes, soft brake. The separated PRP was further diluted (1:25) in HEPES-buffered Tyrode’s buffer containing Cal-520AM (2 μM) and enoxaparin (0.1 mg/mL).

Diluted mouse platelets were incubated at 37^o^C in the dark for 40 minutes. The platelets were then diluted in an equal volume of HEPES-buffered Tyrode’s buffer and incubated at room temperature in the dark for 20 minutes to allow Cal-520–AM deesterification. For all experiments, the labeled platelets were incubated with 1 mM calcium chloride for at least 3 minutes to allow the platelets to recalcify. Subsequently, EGTA (1 mM) was added to chelate residual extracellular calcium.

Thrombin (0.025 U/mL, 0.2 U/mL, and 1 U/mL) and U46619 (5 μM, 10 μM and 20 μM) were added to labeled diluted platelets after the initial baseline recording, and the change in platelet calcium recorded for a further 180 seconds.

### Human platelet calcium flux assay.

Human platelets were isolated from whole blood collected in vacutainers containing Anticoagulant Citrate Dextrose, Solution A (ACD-A) (Becton Dickinson) as previously described (14). The platelet pellet was resuspended in HEPES-Tyrode’s buffer with BSA (0.35% w/v) containing 2 μM fura-2-AM (Invitrogen) and incubated for 45 minutes in the dark. Subsequently, the fura-2-AM–loaded platelets were pelleted by centrifuging at 700*g* for 5 minutes in the presence of platelet inhibitors apyrase (0.02 U/mL, Sigma-Aldrich) and prostaglandin E1 (2 μM, Cayman Chemical). The pellet was resuspended in HEPES-Tyrode’s buffer with BSA and used immediately for calcium flux assays. The human platelets were incubated with 1 mM calcium chloride for 3 minutes immediately prior to the experiment. Then, the platelets were combined with EGTA (2 mM) and ES1 (50 μM) or vehicle and a baseline was recorded for 10 minutes. In other experiments platelets were combined with EGTA (2 mM) and ANX (200 μM) or vehicle for 2 hours, and a baseline was recorded for 72 seconds prior to addition of the SERCA2 inhibitor TG (2 μM) or vehicle control. In other experiments the SERCA3 inhibitor BHQ (10 μM, Sigma-Aldrich) was added instead of TG. The calcium flux was measured for a further 6 minutes after the addition of TG or BHQ or control. Recordings for human platelet calcium were performed using a BMG LabTech CLARIOstar plate reader.

### Platelet protein synthesis assay.

For analysis of de novo platelet protein synthesis, L-AHA (1 μM) was added to washed murine platelets (prepared as described above) resuspended in the HEPES-Tyrode’s buffer and incubated for 8 hours. The platelets were allowed to adhere to a poly-D-lysine–coated (Gibco) Nunc LabTek chamber slide. After platelets were permeabilized, a Click-iT AHA Alex Fluor 488 protein synthesis HCS assay (Invitrogen) was used according to the manufacturer’s instructions to analyze 15–20 platelets per animal to determine the mean L-AHA content per platelet per animal.

### Statistics.

For the lysate proteome, label-free quantification (LFQ) was used, as it was anticipated that the total protein content was unlikely to vary between patients. For the platelet releasate proteome, given that minimal proteins are expected in the resting compared with thrombin-stimulated state, the assumptions required for LFQ analysis were not valid. Therefore, data were normalized against proteins that were minimally changed between the resting and thrombin-stimulated states, which were typically plasma proteins (Supplemental Table 1). Preactivated platelet samples, in which the resting platelet CD62P expression was >20% by flow cytometry, were excluded from the proteomics’ dataset downstream analysis as we have previously described (quality control step) (14).

Data were analyzed using R (v 4.1.2) and visualized using the ggplot2 package and GraphPad Prism 10. Differential expression analyses between (a) DM and non-DM samples or (b) resting and thrombin-stimulated samples were conducted using a moderated 2-tailed *t* test in the limma R package (76). Gene Ontology analysis was conducted using the Gene Ontology Resource (http://geneontology.org/ accessed March 10, 2023) (77).

Wilcoxon’s rank test was used for analysis of samples before and after treatment, such as for ER stress markers after in vitro induction of ER stress in isolated platelets. We used 1-way ANOVA (with Dunn’s post hoc test) and Mann-Whitney tests for comparison of means in nonparametric samples. Two-way ANOVA was used to measure the effect of 2 independent variables on a continuous dependent variable. Welch’s *t* test (2 tailed) was used for comparison of means in parametric data, after checking for normality using Shapiro-Wilk and Kolmogorov-Smirnov normality tests. Fisher’s exact test was used for comparison of proportions between multiple groups. Spearman’s correlation was used to correlate protein quantity and clinical characteristics, given the nonnormal distribution of clinical parameters such as fructosamine, age, body mass index, and coronary artery disease burden. A *P* value less than 0.05 was considered significant.

### Study approval.

Human studies were conducted in accordance with the declaration of Helsinki. Patients provided written informed consent before enrollment. The study was approved by the Sydney Local Health District Human Ethics Committee, Sydney, New South Wales, Australia (protocol no. X20-0085).

Apoe^–/–^ mice were used in accordance with the National Health and Medical Research Council Australia (NHMRC) guidelines and under approval of Sydney Local Health District, Animal Welfare Committee (protocol no. 2020-008). Diversity Outbred hyperglycemic mice and C57BL/6J mice were used in accordance with NHMRC guidelines and under approval of the University of Sydney Animal Ethics Committee (protocol nos. 2017/1274, 2021/1936, 2017/1978).

### Data availability.

Values underlying graphed data and reported means presented in the main text and supplemental material are included in the Supporting Data Values file.

Proteomics datasets have been deposited on the ProteomeXchange Consortium via the PRIDE partner repository and are publicly available (https://www.ebi.ac.uk/pride/archive/projects/PXD049321).

Relevant information about the data may be requested from FHP.

## Supplementary Material

Supplemental data

Supplemental data set 2

Supplemental data set 3

Supplemental data set 4

Unedited blot and gel images

Supplemental video 1

Supplemental video 2

Supporting data values

## Figures and Tables

**Table 1:**
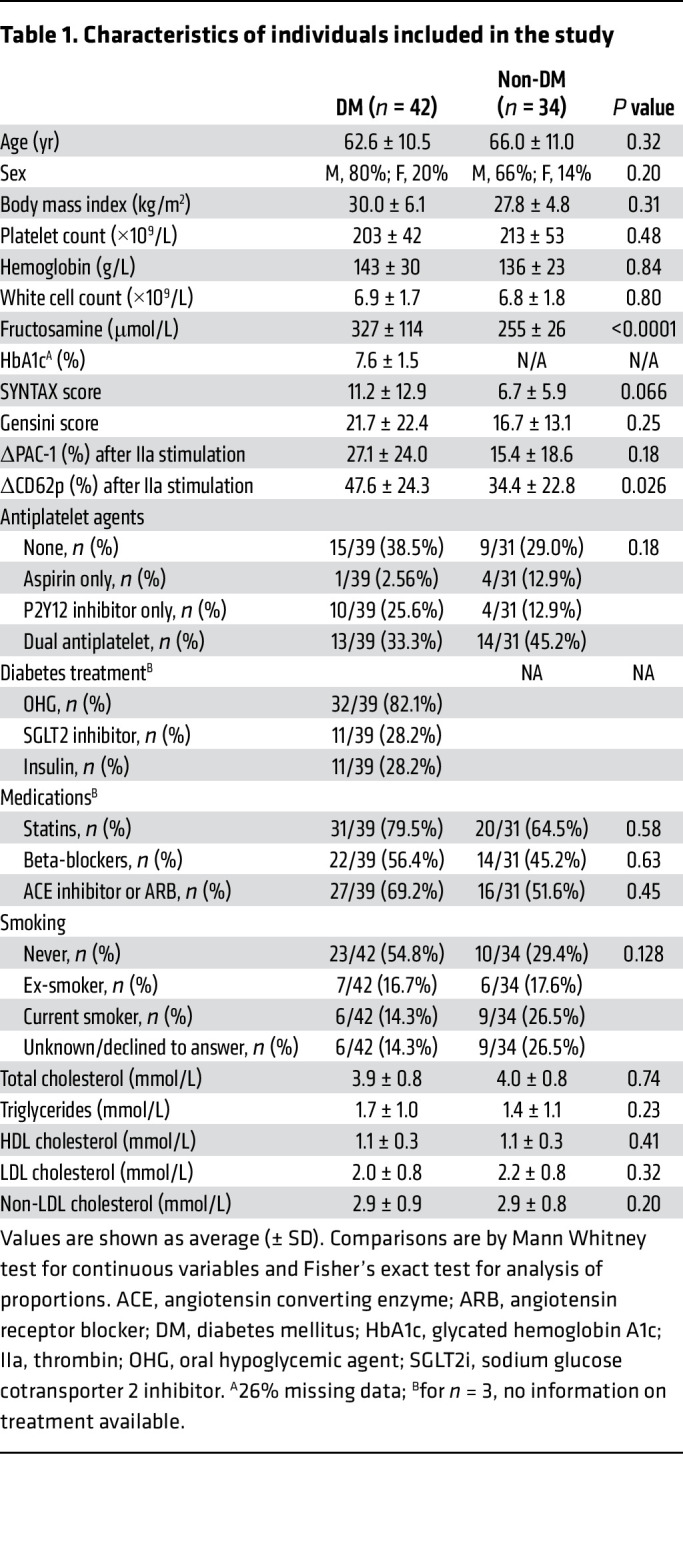
Characteristics of individuals included in the study

**Table 2:**
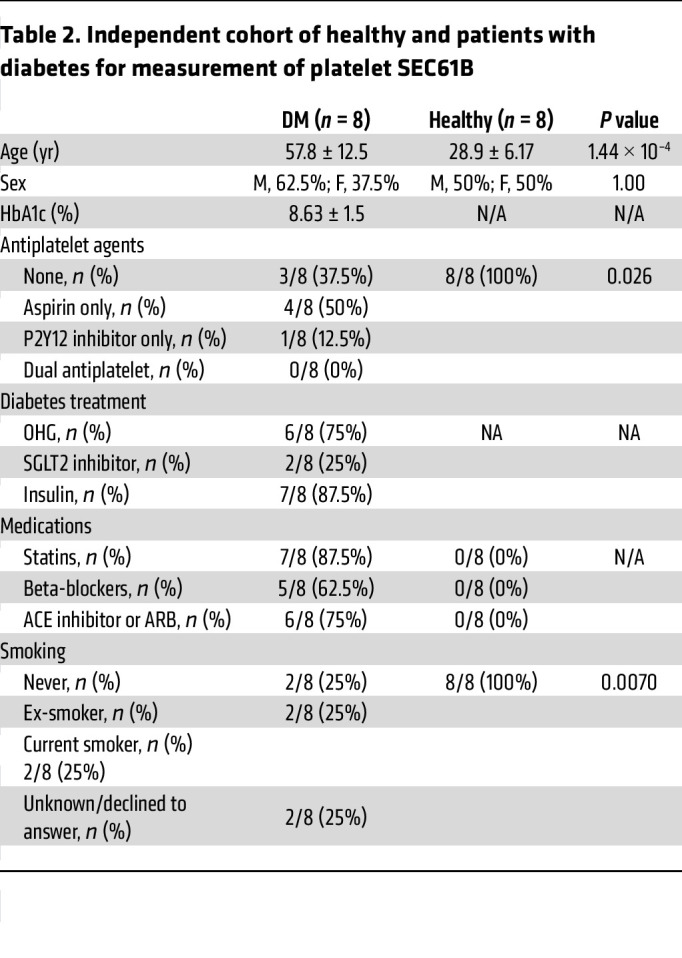
Independent cohort of healthy and patients with diabetes for measurement of platelet SEC61B

**Table 3:**
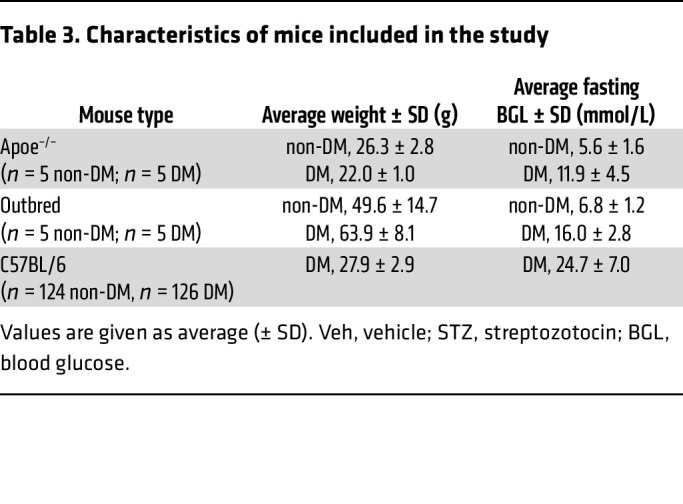
Characteristics of mice included in the study

## Version 1 08/15/2025

Electronic publication
