# Supplementary material for: SEC61B regulates calcium flux and platelet hyperreactivity in diabetes
Source: J Clin Invest. 2025 Aug 15;135(16):e184597. doi: 10.1172/JCI184597 (PMC12352904; doi:10.1172/JCI184597)
Supplement: Supplemental data [file jci-135-184597-s268.pdf]

SUPPLEMENTAL MATERIAL

SEC61B regulates calcium flux and platelet hyperreactivity in diabetes

Yvonne X. Kong[1,2,3], Rajan Rehan[3,4], Cesar L. Moreno[1,5,6], Søren Madsen[1,6], Yunwei Zhang[1,7,8], Huiwen Zhao[1,3], Miao Qi[1,3], Callum Houlahan[1,3,9], Siân P. Cartland[9], Declan Robertshaw[1,3], Vincent Trang[1,3], Frederick Jun Liang Ong[1,3], Michael Liu[1,3], Edward Cheng[1,10], Imala Alwis[1,9], Alexander Dupuy[1,3], Michelle Cielesh[1,10], Kristen C. Cooke[1,6], Meg Potter[1,6], Jacqueline Stöckli[1,6], Grant Morahan[11], Maggie Kalev-Zylinska[12,13], Matthew T. Rondina[14], Sol Schulman[15], Jean Yang[1,7,8], G Gregory Neely[1,5,6], Simone Schoenwaelder[1,9], Shaun Jackson[1,9], David James[1,6], Mary M. Kavurma[9], Samantha Hocking[1,3,16], Stephen M. Twigg[1,3,16], James Weaver[3,4], Mark Larance[1,10]*, Freda H. Passam[1,2,3,9]*

Results

Patient groups with or without diabetes have comparable clinical characteristics

Coronary disease burden was quantified with two different angiographic scoring systems, the SYNTAX and Gensini scores. The Gensini score was 21.7 ± 22.4 in the DM group, and 11.2 ± 12.9 in the non-DM group. The SYNTAX score was 11.2 ± 12.9 in the DM group 6.7 ± 5.9 in the non-DM group. The differences in Gensini and SYNTAX scores were numerically higher in the DM group although not statistically significant. There were no differences in the proportion of patients on single or dual antiplatelet therapies between the DM and non-DM groups (**Table 1**).

Platelet aggregation responses show variability in patients with or without diabetes

Platelet aggregation results are shown in **Supplementary Figure 1**. Response to ADP was supressed in both non-DM and D groups reflecting use of $P2Y_{12}$ antagonists in both groups. Despite the use of aspirin there remained patients with > 50% aggregation in response to arachidonic acid indicative of decreased response to aspirin. Similarly, there were patients with hyperresponsive platelets to low dose thrombin and U46619. Response to collagen was blunted in both groups.

Platelets from patients with diabetes have increased protein secretion in response to low dose-thrombin stimulation

We treated washed platelets from all patients with 0.025U/ml thrombin to elicit potential platelet hyperreactivity to low dose thrombin (**Supplementary Table 2**). Released proteins from DM and non-DM groups after thrombin stimulation included proteins involved in blood coagulation common pathway (GO:0072377), regulation of blood vessel remodelling (GO:0060312) and regulation of platelet activation (GO:0090330).

Methods

Platelet Isolation

For human platelet isolation, we collected blood in 8.5 ml ACD-A tubes (Becton Dickinson, Cat. No. 364606) containing trisodium citrate (22.0 g/L), citric acid (8.0 g/L), dextrose (24.5 g/L) and antimycotic (K sorbate) reagent (0.15 g/L) and is diluted 1:10 in the drawn blood. Additional blood was collected into EDTA (Becton Dickinson, Cat. No. 366643) for full blood count indices and serum tube (Vacuette)

for serum. Platelet isolation and quality control were performed as previously described (1). ACD-A solution was manually removed from the vacutainers and included in the syringe for the blood draw at 10% v/v of mouse blood. After the draw of mouse blood, enoxaparin was added at 40 U/mL.

Platelet aggregation

Platelet aggregation by light transmission aggregometry was performed on 300 µL platelet rich plasma for agonists ADP, arachidonic acid, collagen; and was performed on 300 µL washed platelets (400 x 10^3/µL) in HEPES Tyrode's buffer (129 mM NaCl, 0.34 mM Na2HPO4, 2.9 mM KCl, 12 mM NaHCO3, 20 mM HEPES, 5 mM glucose, 1 mM MgCl2, pH 7.4) for thrombin (IIa) and U46619. Platelet aggregation was recorded for 10 min in response to ADP (5 µM), arachidonic acid (0.5 mg/mL), collagen (2 µg/mL), IIa (0.025 U/ml), U46619 (10 µM) using an AggRAM 1484. Aggregation was determined as the plateau light transmission expressed as % aggregation.

Flow cytometry-based mouse platelet aggregation assay

We performed flow-cytometry-based platelet aggregation for mouse samples as previously described (2) as this allows assessment of platelet aggregation response to agonists with 10 to 25-fold lower sample volumes compared to standard platelet aggregometry. Briefly, whole blood was collected in 10% (v/v) sodium citrate (0.109M, 3.2%) via IVC puncture. Equal volume of HEPES-buffered Tyrodes buffer with enoxaparin (40 U/mL) was added to the whole blood and centrifuged at 240 g at 37°C for 2 min. The platelet rich plasma (PRP) was separated from the red cell bottom layer. PRP was separated into two aliquots of equal volume and incubated

with rat anti-mouse GP1bβ Dylight 488 (Cat. No. X488, Emfret, 1:100) and rat anti-mouse GP1bβ Dylight 649 (Cat. No. X649, Emfret, 1:100), respectively, for 15 minutes at room temperature. After incubation, apyrase (0.02 U/mL) and prostaglandin E1 (2 μM) were added to the PRP and centrifuged for 1 minute at 1960 g. The platelet pellets were resuspended in HEPES-buffered Tyrode's buffer with enoxaparin and diluted to 50 x 10^6/L. Labelled washed platelets were mixed 1:1 and incubated at 37°C for 15 minutes, while shaking at 600 rpm. Platelets were activated with collagen (0.5 -10 μg/mL), ADP (1.25 - 10 μM), thrombin (0.025 - 2 U/mL) and U46619 (2.5 – 40 μM) at 37°C while shaking at 700 rpm for 2 minutes. Samples were fixed by addition of 9x volume of 0.6% (v/v) paraformaldehyde in PBS. Fixed samples were measured by flow cytometry (BD Accuri 6) and analysed with FlowJo software. The number of double-coloured events was quantified as a percentage of the total labelled events (**Supplementary Figure 3**).

Platelet releasate and lysate preparation for proteomic analysis

Double washed platelets (400 x 10^9/mL in pre-warmed (37°C) HEPES-Tyrode's buffer, were divided into "resting control" and "thrombin stimulated" states. Apyrase 0.02 U/mL and human thrombin 0.025 U/mL (Sigma) were added to the resting control and thrombin treated samples respectively. All samples were incubated at 37°C for 5 minutes. PPACK 25 nM (Abcam) was added to terminate thrombin action, and prostaglandin E1 (PGE1) 2 μM was added immediately before centrifugation to obtain the platelet pellet. Platelet-released proteins ("releasate") were in the supernatant, which was removed and stored under argon at -80°C. The platelet pellet was lysed to obtain the intracellular proteins ("lysate") via resuspension in

sodium deoxycholate (SDC) lysis buffer (4% w/v SDC in 0.1 M Tris-HCl, pH 8.0) and stored under argon at -80°C.

Platelet protein preparation for proteomic analysis

Concentration of proteins in the platelet releasate and lysate fractions were quantified using Bicinchoninic Acid (BCA) assay (Thermo Fisher). A total of 5 µg for platelet lysate, and 1 µg for platelet releasate, were processed as previously described for LC-MS/MS analysis (1). RAW proteomics data files were processed using the MaxQuant software (v 1.6.3.4) with reference to the MaxQuant contaminant and the human Uniprot databases. A false discovery rate of 1% using a target-decoy based strategy was used for identification. The MaxLFQ algorithm was used for label-free quantification of proteins as previously described (3). Proteins detected in >50% of samples were included for downstream statistical analysis.

Plasma proteomics analysis

Plasma samples were separated from the platelet poor plasma obtained after platelet isolation. Plasma (1 µL) was added to SDC buffer (24 µL, 1% SDC, 10 mM tris(2-carboxyethyl)phosphine (TCEP), chloroacetamide (40 mM) and Tris-HCl (100 mM, pH 8.5) and heated for 10 minutes at 95°C. Subsequently, samples were left to cool to room temperature, diluted with water, followed by addition of LysC and trypsin (1:100 ratio for protease: protein, µg/µg). The samples were allowed to digest at 37°C for 16 hours and an equal volume of 99% ethyl acetate/1% trifluoroacetic acid (TFA) were added to the digested peptides. Digested peptides were loaded onto SDB-RPS StageTips to extract peptides for downstream analysis by LC-MS, as

previously described (4). Proteins detected in >50% of samples were included for downstream statistical analysis.

Platelet immunofluorescence

C57BL/6J mice were treated with citrate vehicle or streptozotocin (STZ, 55 mg/kg) daily over five consecutive days via intraperitoneal injection. Mice were considered hyperglycemic with a random blood glucose level of >15 mM. Twenty µL of whole blood was collected via tail vein prick into 5 µL of ACD. The whole blood was diluted in 150 µL HEPES-buffered Tyrode's buffer containing apyrase (0.04 U/mL) and centrifuged at 270 g in 37°C for 2 minutes. The supernatant containing the platelets were removed from the red and white cell pellet. This process was repeated by resuspending the pellet in HEPES-Tyrode's buffer. The supernatants were combined before being aliquoted into poly-D-lysine (0.1 mg/mL) coated LabTek 8-well chamber slides. The platelets were allowed to adhere for at least one hour prior to fixation with 2% (v/v) paraformaldehyde. The platelets were washed with 3% BSA (w/v) prior to permeabilization with Triton-X100 (0.5%) in PBS for 20 minutes. Wells were blocked with 1% BSA (w/v) in PBS for at least one hour prior to incubation with rabbit anti-SEC61B (Cat. No.14648S, Cell Signaling Technology, 1:200 dilution), rabbit phospho-IRE1 alpha (Ser724) polyclonal antibody (Cat. No. PA5-105424, Thermo Fisher, 1:100 dilution), or GRP78 (Cat. No. ab21685, Abcam, 1:200 dilution) antibodies. Platelets were highlighted with anti-mouse GP1bβ-Dylight 488 (1:200). Secondary goat anti-rabbit IgG Alexa Fluor 647 (Cat. No. A-21245, Invitrogen, 1:1000 dilution) was used to detect unlabelled rabbit primary antibodies. Imaging was performed using a Zeiss 880 confocal microscope with an 100x oil objective. Fifteen to 20 platelets were analysed per animal using ImageJ analysis software,

with the total target protein signal per platelet minus the signal in platelets without primary antibody, but with the secondary antibody, reported.

***In vitro* induction of platelet endoplasmic reticulum (ER) stress and platelet flow cytometry**

Washed platelets (400 x 10^6/mL) were treated with thapsigargin (2 μM) or BHQ (10 μM) for 1 hour or equal volume of DMSO control for in vitro induction of ER stress. Aliquots of the treated platelets were then analysed by flow cytometry for surface CD62P (Cat. No. 550888, BD Biosciences), PAC-1 positivity (Cat. No. MA528564, Thermofisher Scientific), ERp5 AF488 (G-5 clone, Cat. No. SANTSC-365260 P, Santa Cruz Biotechnology) and PDI AF647 (Cat. No. ab20282, Abcam). A BD Accuri 6 flow cytometer was used. The platelets were subsequently pelleted, lysed in RIPA lysis buffer with protease inhibitor cocktail (Sigma Aldrich) and phosSTOP (Roche). The supernatant was resuspended in Laemmlli buffer with beta-mercaptoethanol and heated for 10 min at 90°C for Western Blot analysis (**Supplementary Figure 6**). Platelet releasate or lysate samples were resolved on 4-20% polyacrylamide gels (Bio-Rad) under reducing conditions with beta-mercaptoethanol and transferred onto polyvinylidene difluoride (PVDF) membranes using the IBlot2 Dry Blotting system (Thermo Fisher). PVDF membranes were blocked in 1% bovine serum albumin (BSA) in tris buffered saline-0.1% v/v Tween (TBS-T). The blots were incubated separately with primary antibodies overnight before secondary staining using a horse-radish peroxidase (HRP) secondary antibody (goat anti-rabbit IgG H&L HRP, Cat. No. ab97051, Abcam, 1:2000 dilution, rabbit anti-mouse IgG HRP, Cat. No. P026002-2, Agilent, 1:1000 dilution). Primary antibodies used were the anti-phospho-IRE1 antibody (1:1000), IRE-1 rabbit mAb (14C10) (Cat. No. 3294S, Cell

Signaling Technology, 1:1000), the anti-SEC61B Ab (1:1000), anti-Sec61A1 (D7Q6V) rabbit mAb (Cat. No. 14868, Cell Signaling Technology, 1:1000), rabbit polyclonal anti-EIF2S1 (eIF2a) (phospho S51) antibody (Cat. No. ab131505, Abcam, 1:1000), anti-eIF2α Antibody (Cat. No. 9722, Cell Signaling Technology, 1:1000), anti-GAPDH (Cat. No. MA515738, Thermo Fisher, 1:4000), Beta-actin antibody 4967S (Cat. No. 4967S, Cell Signaling Technology, 1:1000).

Bone marrow immunofluorescence

Bilateral femurs from Apoe-/- and outbred mice were carefully dissected and fixed in 2% (v/v) paraformaldehyde for 24 hours. The femurs were decalcified in ethylenediaminetetraacetic acid (EDTA) 0.5M for 48 hours and dehydrated in sucrose (30% w/v) before being mounted in O.C.T. and stored at -80 ^{o}C. Ten µm sections of bone marrow were obtained for immunofluorescence. Sections were blocked with foetal bovine serum (FBS, 10%), Triton-X100 (0.16%) in PBS for at least one hour prior to staining. Sections were stained with rabbit anti-phospho-IRE1 Ab (1:100), rabbit anti-p-eIF2a (1:200), anti-SEC61B Ab (1:200), and rabbit anti-GRP78 (Cat. No. ab21685, Abcam, 1:200 dilution). Goat anti-rabbit IgG antibody conjugated to Alexa Fluor 647 (Cat. No. A-21245, Invitrogen, 1:1000) was used to identify these markers. Megakaryocytes were identified by staining with anti-mouse GP1bβ-Dylight 488 (1:200) and nuclei were visualised by staining with Hoechst 33258 (1:10,000). Imaging was performed using a Zeiss 880 confocal microscope with 40x water or 63x oil objectives. Images were taken to obtain 15-20 megakaryocytes per bone marrow sample. Image analysis was performed using ImageJ, with the mean intensity in the region of interest minus the mean intensity of

megakaryocytes without primary antibody, but with secondary antibody, reported (**Figure 4** and **Supplementary Figure 2**)

Generation of *SEC61B* knockout and overexpressing HEK293 cells

CRISPR knockouts were generated using the lentiCRISPRV2 vector (Addgene). HEK293 cells were from American Type Culture Collection, Manassas, VA, USA). HEK293 cells were tested every 2-3 months for mycoplasma and were negative. Lentiviruses carrying targeting sgRNAs or controls were packaged by co-transfection with pCAG-VSVG, and psPAX2 and the vector of interest at a ratio of 1:3:3 using lipofectamine 3000 in HEK293 cells. Virus was collected and purified from the media by centrifugation. For virus transduction, HEK293 cells were plated at 20% confluence, and exposed to the respective virus in the presence of polybrene (8 µg/ml). Cells were then selected using puromycin (2 µg/ml) for a period of 3 days. The sgRNAs sequences used for generating KO1 and KO2 were (i) 5'-ACCCCCAGTGGCACTAACGT-3' and (ii) 5'-GTAGAATCGCCACATCCCCC-3', respectively. The control sgRNA sequenced used was 5'-GCGTCTGAGATGAGAAAAGAT-3'.

The ORF for the Human *SEC61B* (NM_006808) was purchased from Origene (RG200247) and cloned using NEBuilder HiFi DNA Assembly (NEB #E2621) into a lentiviral backbone (Addgene, cat# 52961) for stable expression. The following primers were used to amplify the recipient vector (i) 5'- atgaccgagtacaagcccac-3' and (ii) 5'-GGTACCTTAATTAACCAAACTGGATC-3'. Two fragments were amplified and inserted for rational vector design. Fragment 1, *SEC61B* ORF, was amplified using these primers (i)

5'- ATCCAGTTTGGTTAATTAAGGTACCTGAATCAATATTGGCAATTAGCC

-3' and (ii) 5'- GGACAGTGCCAAGCAAGCA-3'; Fragment 2, *Ef1a* promoter, was amplified from Addgene 52961, using 5'-TTGAGTTGCTTGCTTGGCACTGTCCttttttgaattcgctagctaggtcttgaaag-3' and 5'-cgcaccgtgggcttgtactcggtCATatggtggcagcgctctagaac-3'. Control viruses without *SEC61B* ORF insert were also generated. Viruses were packaged in HEK293 cells with psPAX2 (Addgene), pCAG-VSVg (Addgene) using Lipofectamine 3000 Transfection Reagent (Thermo Fisher Scientific). HEK293 cell lines were infected in the presence of polybrene (8 µg/ml) and selected for 1 week in puromycin (2 µg/ml) before proceeding to experiments.

HEK293 cells immunofluorescence

Nunc Lab-Tek II Chamber Slides were coated with fibronectin (25 µg/mL) overnight followed by removal of fibronectin and washing with PBS. Cells were added (75,000/well) and allowed to grow for 48 hours. They were then washed and fixed with 4% PFA for 15 min, washed x 2 with PBS 3% BSA, permeabilized with 0.5% Triton X-100 for 20 min at room temperature. Primary Ab (SEC61B 1:200 or equal concentration of rabbit IgG control) was incubated with the cells for 1 hr at room temperature. Cells were washed x 3 with PBS 3% BSA. Secondary antibody was added for 2h at room temperature; anti-rabbit AF647 (Cat. No. A-21245, Invitrogen, 1 :1000), anti-tubulin-488 (Cat. No. 322588, Invitrogen, 1:250), and phalloidin-AF594 (Cat. No. A12381, Invitrogen, 1:400). The antibody was removed, cells were washed x 3 and incubated with Hoescht 33342 1:2000 for 10 min at RT. Cells were washed again with PBS x 3, then imaged. Fluorescence intensity was quantified for 30-40 cell clusters per genotype from n=3 independent experiments. Image analysis was performed using ImageJ, with the mean intensity in the region of interest, minus the

mean-intensity of cells stained with control IgG, normalized to the fluorescence intensity of control HEK293 cells.

HEK293 calcium flux assays

HEK293 cells, with SEC61B overexpression or control, were plated onto fibronectin-coated (25 µg/mL) Nunc LabTek 8-well chamber slides at 75,000 cells per well and allowed to recover overnight. The cells were incubated with Cal-520-AM (Abcam) 2µM, probenecid (Sigma-Aldrich) 2 mM and Pluronic F127 (Sigma-Aldrich) 0.1% v/v final concentration, in phenol-red free DMEM supplemented with 10% FCS for 30 minutes at 37°C, 5% CO_2. The Cal-520-AM loading solution was removed and replaced with fresh phenol-red free media supplemented with 10% FCS, and cells were further incubated for 15 minutes at 37°C, 5% CO_2 to allow the Cal-520-AM to de-esterify. The medium was then removed, and cells washed with calcium free imaging buffer (125 mM NaCl, 2 mM $MgCl_2$, 4.5 mM KCl, 10 mM glucose, 20 mM HEPES, pH 7.4). Eeyarestatin I (ES1, Cayman Chemical) 10 µM, which captures SEC61 complex in a Ca^{2+}-permeable state (5), was added immediately prior to the calcium flux assay to permit the translocon-mediated calcium leak from the ER (1x volume). Calcium mobilisation from the ER to the cytosol was induced by addition of 3x volume of thapsigargin (Merk Life Science), dissolved in imaging buffer at a final concentration 2 µM, after 75 seconds of baseline readings. Cells were imaged with a Zeiss 880 confocal microscope with 20x lens using the "Time Course" function with images recorded every second. The data were analysed with ImageJ using the "Time Series Analyzer" plugin. A total of 4-6 regions of interest were identified per experiment, and these were used to generate the mean Cal-520 signal per experiment. The mean Cal-

520 signal for each experiment were used to generate the time course and for the peak Cal-520 signal analysis.

HEK293 cells, with *SEC61B* knockout or control, were seeded into poly-D-lysine (Thermo Fisher) coated 96 well clear bottom black plates at 75,000 cells per well. The cells were allowed to recover for ~24 to 48 hours prior to the calcium flux assays. HEK293 cells were incubated with fura-2-AM (Thermo Fisher) 2 µM with pluronic F127 (0.1% v/v final concentration) and probenecid 2 mM in phenol red free DMEM (Gibco) supplemented with 10% FCS and GlutaMAX 1x (Gibco) for 60 minutes at 37°C, 5% CO2. The fura-2-AM loading solution was removed and replaced with fresh phenol red free media supplemented with 10% FCS and cells were further incubated for 30 minutes to allow for fura-2-AM de-esterification. Calcium mobilization from the ER to the cytosol was induced by automated injection of thapsigargin (final concentration 2 µM), mixing by automated double-orbital function after 90-120 seconds of baseline readings (**Supplementary Figure 5**).
In other experiments, calcium flux was measured with Cal-520 in the absence of probenecid (**Supplementary Figure 5**).
Separately, HEK293 control or OE cells were incubated with vehicle or anisomycin (ANX) 200 µM for 2h, followed by measurement of calcium flux with Fura 2 340/380 nm, as above (**Supplementary Figure 2C**).
The fluorescent readings were conducted using the BMG Labtech CLARIOstar Plus with the cytosolic calcium represented by the ratio of fluorescent emission at 510 nm, after excitation at 340 nm and 380 nm.

HEK293 protein synthesis assay and western blots of ER stress markers

HEK293 cells with *SEC61B* overexpression or knockout, and their respective controls, were seeded onto poly-D-lysine coated Nunc Lab-Tek II chamber slides at 75,000 cells per well. The cells were allowed to recover for ~24 hours prior to the protein synthesis assay. The Click-iT AHA Alex Fluor 488 protein synthesis HCS assay (Invitrogen) was used according to the manufacturer's instructions, with the alteration that cells were incubated with media containing L-azidohomoalanine (L-AHA) for 1 hour. Cells were imaged using a Zeiss 880 confocal microscope with 100x oil objective. Images were taken to obtain ~30 cellular clusters per genotype. Image analysis was performed using ImageJ, with the mean intensity in the region of interest, minus the mean-intensity of cells treated with media without L-AHA, reported (**Figure 5F** and **Supplementary Figure 5**).

HEK293 cell (control and OE) lysate protein concentrations were estimated with a BCA Protein Assay Kit (Pierce), and 20 μg of protein were prepared under reducing conditions (with 10% (v/v) 2-mercaptoethanol) and resolved on 4-20% (w/v) polyacrylamide gels. Proteins were transferred onto a PVDF membrane using the iBlot2 Dry Blotting System (Invitrogen). Membranes were blocked for 1 hr with 3% (w/v) BSA in TBS buffer containing 0.1% (v/v) Tween 20, then incubated overnight at 4°C with primary antibody p-IRE1, IRE1, GAPDH (**Figure 5B-E**), p-eIF2a, eIF2a, or b-actin (**Supplementary Figure 4A-B**) diluted 1:1000 in the blocking solution, followed by incubation for 1 hr with HRP-conjugated secondary antibody diluted 1:1000 in the blocking solution. Chemiluminescent protein detection with ECL Western Blotting Substrate (Pierce) was performed in a ChemiDoc MP imaging system (BioRad). Protein band intensities were measured using the Image Lab software (BioRad) volume analysis tool.

Platelet adhesion under flow

Microfluidic channels (100 μm height, 1000 μm width) were dry-bonded onto #1 borosilicate coverslips at 60 degrees Celsius for 15 min. Channels were then coated with human fibrinogen at 60 μg/mL (Haematologic Technologies) for 2 hrs at room temperature, washed with PBS. Citrated whole blood was treated with either vehicle (0.1% v/v DMSO) or anisomycin (Calbiochem) at 200 μm for 2 hrs at room temperature, stained with Calcein AM at 1 ug/mL (Thermofisher), then perfused through microfluidic devices at 1000 s^{-1} for 5 min. Adhesion of platelets were visualized by excitation with a CoolLED pE-800 illumination system and imaged using an Olympus IX81 microscope on a 40x objective (NA 1.2). Surface area coverage was determined by measuring the area of fluorescent cells adherent to the surface of the coverslip and thresholded against background fluorescence (6) (**Supplementary Figure 4**).

Needle in situ model and intravital microscopy

All mice used in this model were pretreated with anisomycin, or an equivalent volume of DMSO, by intravenous tail vein injection, 90 minutes prior to start of surgery. Anisomycin was used at 20 μg/g body weight (to max dose of 530 μg), consistent with previous studies (7). Thrombi were induced in the mesenteric venules of DM and non-DM mice using the needle in situ mode (8). Microinjector needles were fashioned using glass capillaries (GD-1, 50 mm; Narishige) with a micropipette puller (P-1000; Sutter Instruments, USA) such that the needle tip had a diameter of 2 - 3 μm. The needle was inserted into the lumen of mesenteric vessels (100 - 200 μm in diameter), with the needle tip positioned ~20 μm from the base of the vessel using an Injectman micromanipulator (Eppendorf, Hamburg, Germany).

Thrombus formation at the needle tip was monitored for 4 minutes under real-time DIC microscopy using an Olympus IX81 inverted microscope (X40 objective, UPlan NA:1.3) and footage recorded via a sCMOS camera (pco.edge 4.2 bi; Excelitas, Kelheim, Germany) for off-line analysis using Visiview software 2.1.4 (Visitron System GmbH, Germany). Thrombus surface area at the indicated times was determined using ImageJ (Ver 1.48v) software.

References

1. Houlahan CB, et al. Analysis of the healthy platelet proteome identifies a new form of domain-specific O-fucosylation. *Mol Cell Proteomics.* 2024;100717.

2. De Cuyper IM, et al. A novel flow cytometry-based platelet aggregation assay. *Blood.* 2013 Mar 7;121(10):e70-80.

3. Cox J, et al. Accurate proteome-wide label-free quantification by delayed normalization and maximal peptide ratio extraction, termed MaxLFQ. *Mol Cell Proteomics.* 2014;13(9):2513-2526.

4. Harney DJ, et al. Proteomic Analysis of Human Plasma during Intermittent Fasting. *J Proteome Res.* 2019;18(5):2228-2240.

5. Gamayun I, et al. Eeyarestatin compounds selectively enhance Sec61-mediated Ca2+ leakage from the endoplasmic reticulum. *Cell Chem Biol.* 2019;26(4):571-583.

6. Dupuy A, Ju LA, Passam FH. Straight Channel Microfluidic Chips for the Study of Platelet Adhesion under Flow. *Bio Protoc.* 2019;9(6):e3195

7. Park GL, et al. Anisomycin protects against sepsis by attenuating IκB kinase-dependent NF-κB activation and inflammatory gene expression. *BMB Rep.* 2021;54:545-550.

8. Ju L, et al. Compression force sensing regulates integrin alpha(IIb)beta(3) adhesive function on diabetic platelets. *Nat Commun.* 2018;9(1):1087.

Supplementary Table 1. Proteins used as controls for normalisation of platelet releasate proteins.

A1BG	APOC3	CA1	HPR	IGJ	KRT9	SERPINA6
A2M	APOD	CD9	HPX	IGKC	KRT10	SERPINC1
ACTC1	APOE	CD36	IGFALS	IGKV2-40	KRT14	SERPIND1
AFM	APOL1	CP	IGHA1	IGKV3D-11	KRT16	SERPING1
AGT	ARF1	DCD	IGHG1	IGKV3D-20	ORM1	SLC4A1
ALB	ARPC2	FGA	IGHG2	IGKV4-1	ORM2	SPTLC2
AMBP	AZGP1	FGB	IGHG3	IGLL5;IGLC1	PARVB	STOM
APOA1	BIN2	FGG	IGHG4	ITGB3	PON1	SYNE1
APOA2	C4A	GC	IGHM	KRT1	PPIB	TF
APOA4	C4BPA	GP1BA	IGHV3-15	KRT2	RAP1B	TUBB
APOC1	C5	HBA1	IGHV3-66	KRT5	RSU1	
APOC2	C9	HP	IGHV3-72	KRT6A	SERPINA3	

Supplementary Table 2. Proteins significantly released by DM and non-DM platelets after stimulation with low dose thrombin (0.025U/mL).

non-DM

protein	log2fold	protein	log2fold
**B4GALT1 ****	3.02	FSTL1	1.96
CTGF	2.98	CTSW	1.95
ANGPT1	2.76	THBS1	1.93
CLEC11A	2.66	CST3	1.92
XYLT2	2.55	SRGN	1.90
GLG1	2.52	SEC23IP	1.88
APLP2	2.50	LTBP1	1.86
QSOX1	2.49	TGFB1	1.86
MAN1A1	2.46	ANG	1.81
CXCL3	2.42	CCL5	1.76
SERPINE1	2.36	F5	1.73
TFPI	2.34	SPARC	1.70
EFEMP1	2.30	VWF	1.69
LOXL3	2.30	PROS1	1.68
DNAJC3	2.25	PSAP	1.67
MAN2A1	2.23	DAG1	1.65
NID2	2.21	MMRN1	1.60
APP	2.18	NUCB1	1.58
PCSK6	2.18	DPP7	1.54
PDGFA	2.17	EMILIN1	1.53
VEGFC	2.15	CTSA	1.50
FAM3C	2.13	HPSE	1.48
PDGFD	2.12	ECM1	1.48
CXCL5	2.10	SPP2	1.40
CALU	2.05	RARRES2	1.35
SERPINE2	2.02	**CPB2 ****	1.34
NID1	2.02	CTSD	1.32
**TIMP1 ****	1.99	**LGALS3BP ****	1.30
PF4	1.98	TPP1	1.28
PDGFB	1.98	PPBP	1.23
IGF2	1.96		

DM

protein	log2fold	protein	log2fold
CTGF	2.98	**BDNF ***	1.68
ANGPT1	2.76	PSAP	1.67
CLEC11A	2.66	**ISLR ***	1.67
XYLT2	2.55	DAG1	1.65
GLG1	2.52	**GLCE ***	1.64
APLP2	2.50	MMRN1	1.60
QSOX1	2.49	NUCB1	1.58

MAN1A1	2.46	**RNASET2 ***	1.55
CXCL3	2.42	**ADAMDEC1 ***	1.55
SERPINE1	2.36	**GALNT1 ***	1.55
TFPI	2.34	DPP7	1.54
EFEMP1	2.30	EMILIN1	1.53
LOXL3	2.30	**SORT1 ***	1.51
DNAJC3	2.25	CTSA	1.50
MAN2A1	2.23	**AQR ***	1.48
NID2	2.21	HPSE	1.48
APP	2.18	ECM1	1.48
PCSK6	2.18	**TPMT ***	1.48
PDGFA	2.17	SPP2	1.40
VEGFC	2.15	**TGFBI ***	1.39
**CLSTN1 ***	2.15	**SDC4 ***	1.38
FAM3C	2.13	**COCH ***	1.37
PDGFD	2.12	**TNXB ***	1.35
**TIMP3 ***	2.12	RARRES2	1.35
**PCOLCE ***	2.12	**MAN1B1 ***	1.33
CXCL5	2.10	**GALNT2 ***	1.33
CALU	2.05	CTSD	1.32
SERPINE2	2.02	**CSDE1 ***	1.30
NID1	2.02	**CTSC ***	1.29
TIMP1	1.99	**GOLIM4 ***	1.29
SPTA1 *	1.99	**UXS1 ***	1.28
PF4	1.98	TPP1	1.28
PDGFB	1.98	PPBP	1.23
IGF2	1.96	FGB	1.23
FSTL1	1.96	FGA	1.21
CTSW	1.95	ESAM	1.20
**RNASE4 ***	1.95	**C8B ***	1.18
THBS1	1.93	FGG	1.16
CST3	1.92	**PLXDC2 ***	1.14
SRGN	1.90	**FUT8 ***	1.09
SEC23IP	1.88	**PLOD3 ***	1.08
LTBP1	1.86	**CFHR2 ***	1.06
TGFB1	1.86	**CLU ***	1.03
**DCN ***	1.83	GP5	1.00
ANG	1.81	**LAMC1 ***	0.99
CCL5	1.76	**NUCB2 ***	0.96
F5	1.73	**TREML1 ***	0.96
SPARC	1.70	**LYZ ***	0.87
**IGFBP2 ***	1.70	**SIAE ***	0.86
VWF	1.69	**FN1 ***	0.85
PROS1	1.68	**GGH ***	0.69
**BDNF ***	1.68	**PLG ***	0.69
PSAP	1.67	**MAP3K5 ***	0.58
PROS1	1.68		

* Indicates proteins that are uniquely released by DM platelets after low dose (0.025U/mL) thrombin stimulation; ** indicates proteins uniquely released by non-DM platelets after low dose thrombin stimulation.

Supplementary Figure 1

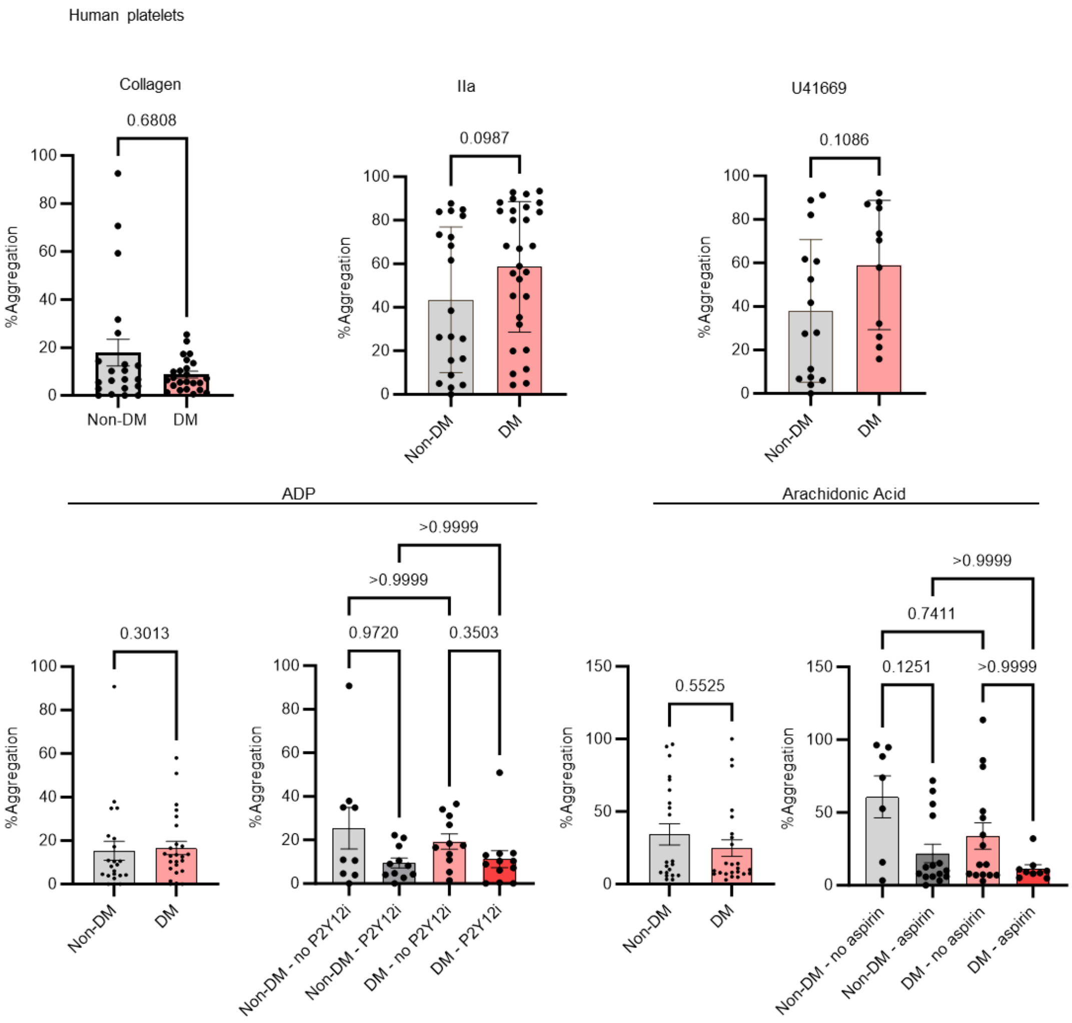

Supplementary Figure 1. Platelets from patients with type 2 diabetes show variability in their aggregation responses to agonists both on and off antiplatelet treatment. Platelet aggregation expressed as % of non-DM (grey) and DM (red) platelets in response to platelet agonists collagen 2 µg/ml, thrombin (IIa) 0.025 U/ml, U46619 10 µM, ADP 5 µM, and arachidonic acid 0.5 mg/mL. Aggregation to ADP and arachidonic acid shown for patients with and without DM, receiving or not receiving P2Y12 inhibitor (P2Y12i) or aspirin, respectively. Mann-Whitney test. U46619=thromboxane A2 receptor agonist.

Supplementary Figure 2

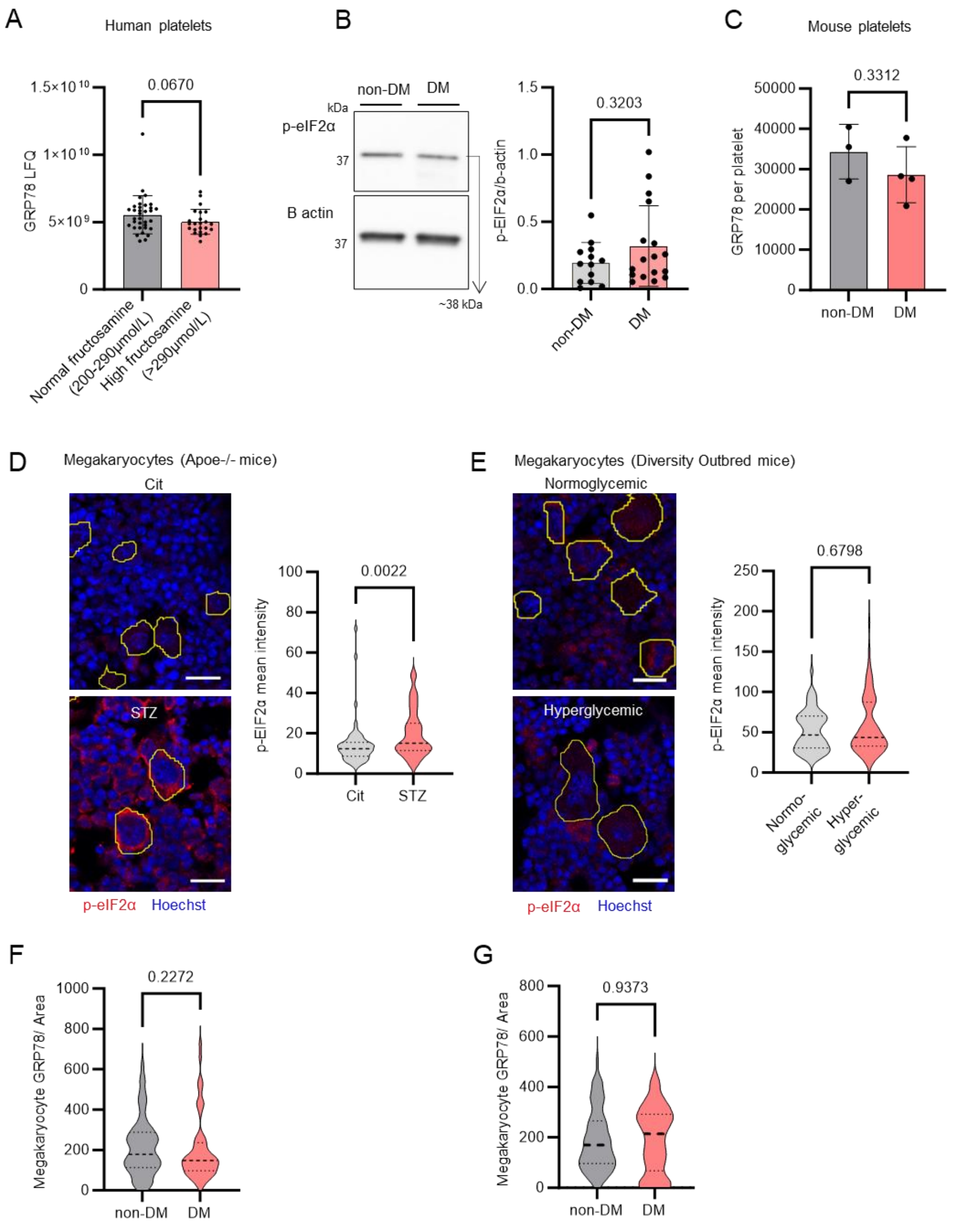

Supplementary Figure 2. GRP78 and p-eIF2a (PERK pathway) is not increased in diabetic platelets. A. GRP78 LFQ in platelet lysate from patients with normal fructosamine (200-290 µM) versus high fructosamine (>290 µM), Welch's t-test. **B.** Representative western blots of platelet lysate p-eIF2a and beta actin from non-DM

and DM patients. P-eIF2a/actin ratio in non-DM and DM samples, mean ± SD, Mann-Whitney test. **C.** Average GRP78 fluorescence intensity of immunostained platelets from normoglycemic (Veh, n=3, blue) and hyperglycemic (STZ, n=4, red) mice, Welch's t-test. **D.** Immunostaining of p-eIF2a in megakaryocytes of non-DM (Cit) and DM (STZ) Apoe-/- mice. Representative images. Immunofluorescence intensity of p-eIF2a in non-DM Apoe-/- mice (Veh, grey) versus DM mice (red), Mann-Whitney test**. E.** Immunostaining of p-eIF2a in megakaryocytes of normoglycemic and hyperglycemic diversity outbred mice. Representative images. Immunofluorescence intensity of p-eIF2a in normoglycemic (Veh, grey) versus hyperglycemic mice (red), Mann-Whitney test. The scale bar is 20 µm. **F.** Immunofluorescence intensity of GRP78 in non-DM Apoe-/- mice (Veh, grey) versus DM mice (red), n~15 megakaryocytes per mouse, and **G.** non-DM outbred mice (grey) versus DM outbred mice (red); n=15-20 megakaryocytes per mouse. Mann-Whitney test.

Supplementary Figure 3

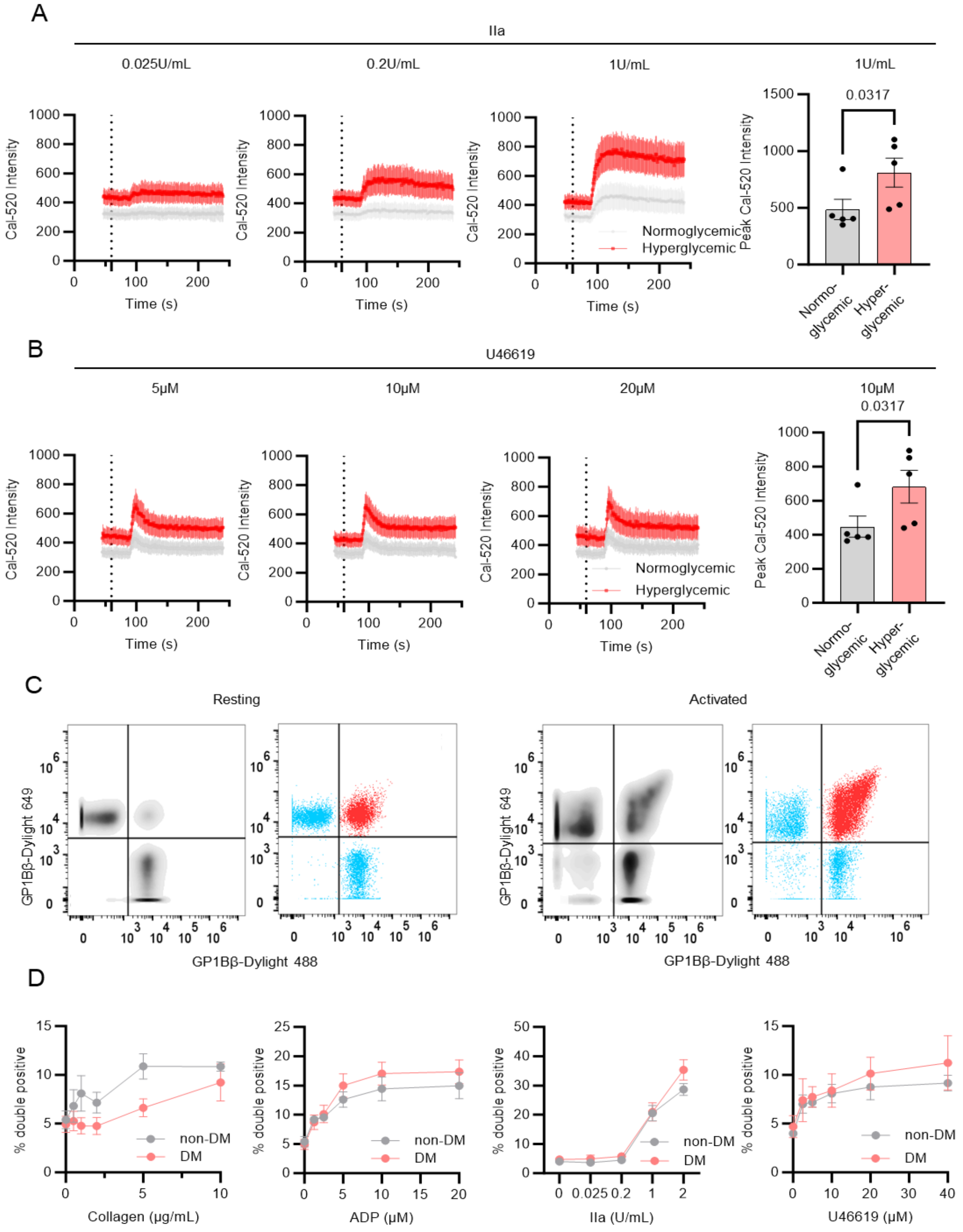

Supplementary Figure 3. Platelets from hyperglycemic mice have increased calcium flux in response to thrombin and U46619. A. Time course of cytosolic

calcium changes, measured by Cal-520, in platelets isolated from non-DM (grey) and DM (red) mice after addition of thrombin (IIa) at doses of 0.025, 0.2, and 1 U/ml. Peak cytosolic calcium in non-DM (grey) and DM (red) platelets after addition of thrombin at 1 U/ml. Mean ± SD, n=5 mice per group, Mann-Whitney test. **B.** Time course of cytosolic calcium changes, measured by Cal-520, in platelets isolated from non-DM (grey) and DM (red) mice after addition of U46619 at doses 5, 10, and 20 μM. Peak cytosolic calcium in non-DM (grey) and DM (red) platelets after addition of U46619 at 10 μM. Mean ± SD, n=5 mice per group, Mann-Whitney test. **C.** Gating strategy for the measurement of platelet aggregates by mixing 2 suspensions of platelets labelled with GPIBb conjugated to different fluorophores (GPIBb-Dylight 649 and GPIBb-Dylight 488) followed by addition of agonist. Platelet aggregation was quantified by gating on the double positive platelet population (red) whereas non-aggregating platelets were detected by single positivity (blue). Scatter plots of mixed platelets without addition of agonist (resting) are shown on the left panel. Scatter plots of mixed platelets with the addition of agonist are shown on the right panel. **D.** Dose curve of % double positivity after addition of collagen (0.5 - 10 μg/ml), ADP (1.25 – 20 μM), IIa (0.025 – 2 U/ml), and U46619 (2.5 - 40 μM) to platelets isolated from non-DM and DM mice. Mean ± SEM, n=3-4 mice per group for each agonist, 2 way-ANOVA.

Supplementary Figure 4

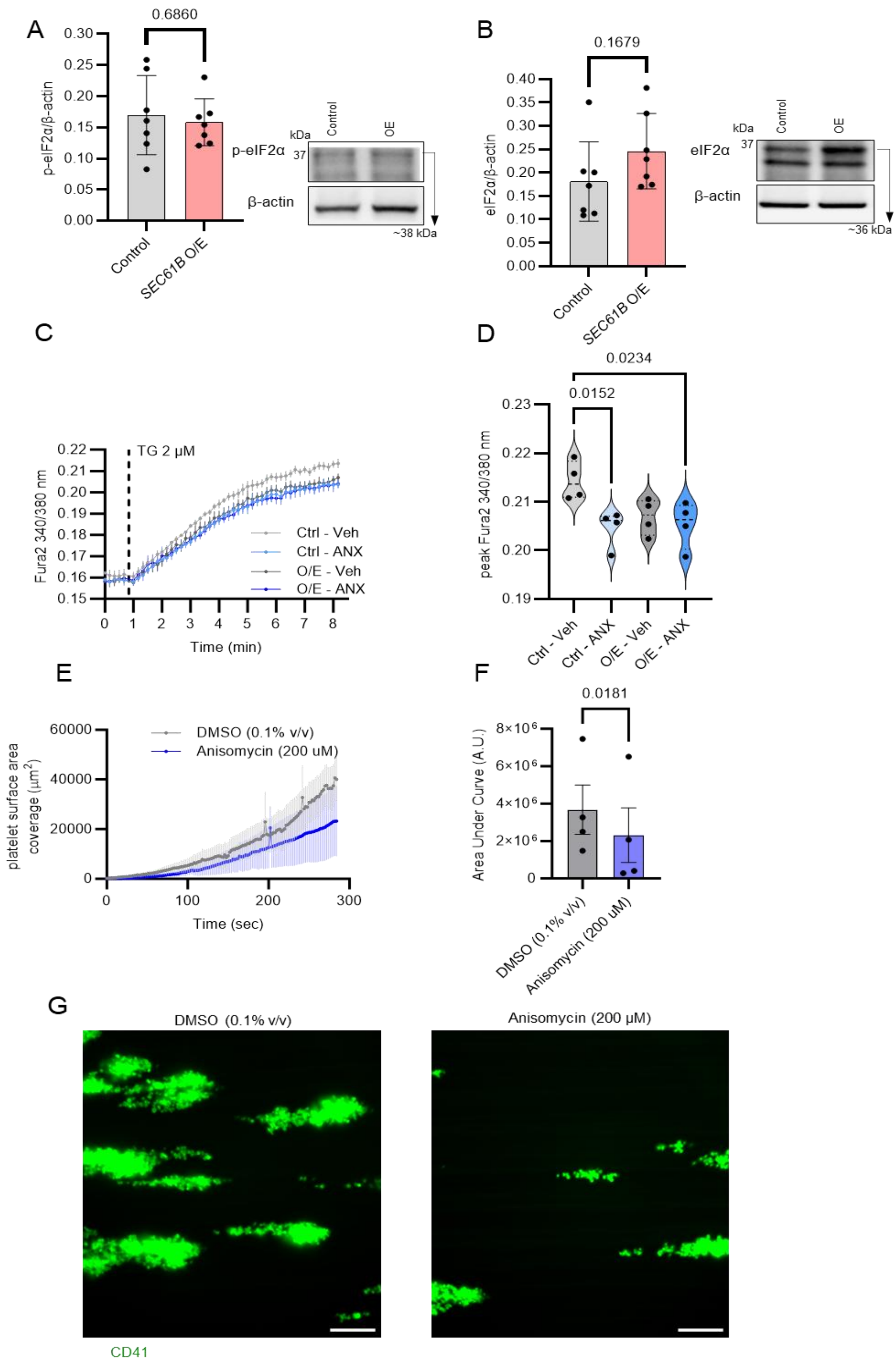

Supplementary Figure 4. SEC61B overexpression does not increase activation of eIF2a (PERK pathway). Anisomycin inhibits calcium flux in WT HEK293 cells

and inhibits platelet adhesion to fibrinogen matrix. A. Representative western blots of p-eIF2a, **B.** eIF2a, and beta actin in HEK293 control and *SEC61B*-overexpressing cells (OE). Band intensity ratio of p-eIF2a or eIF2a to actin in HEK293 lysate of control (grey) versus OE cells (red), n=7 independent experiments, unpaired t test. **C.** Average fluorescence intensity of Fura2 340/380 nm of control (grey) and SEC61B-OE (blue) over time in the presence of vehicle control (Veh) or ANX, followed by treatment with TG (solid line=mean; shaded region=SEM). **D.** Peak fluorescence intensity of Fura2 after addition of TG in control (grey) and OE cells (blue) from n=4 independent experiments per genotype, one-way ANOVA with Dunnett's multiple comparisons test. **E.** Average platelet fluorescence surface area coverage on a fibrinogen coated microfluidic channel after perfusion of whole blood treated with ANX 200 µM for 2 h or vehicle at a shear rate of 1000 s^{-1} for 5 min. Solid line=mean; shaded region=SEM. **F.** Area under curve of platelet fluorescence over time after perfusion of blood treated with vehicle (grey) or ANX 200 µM blue. n=4 separate healthy donors, paired t-test. **G.** Representative images of platelet fluorescence area (green) after perfusion of blood treated with vehicle (left) or ANX (right) on fibrinogen-coated microfluidics channels. The scale bar is 50 µm.

Supplementary Figure 5

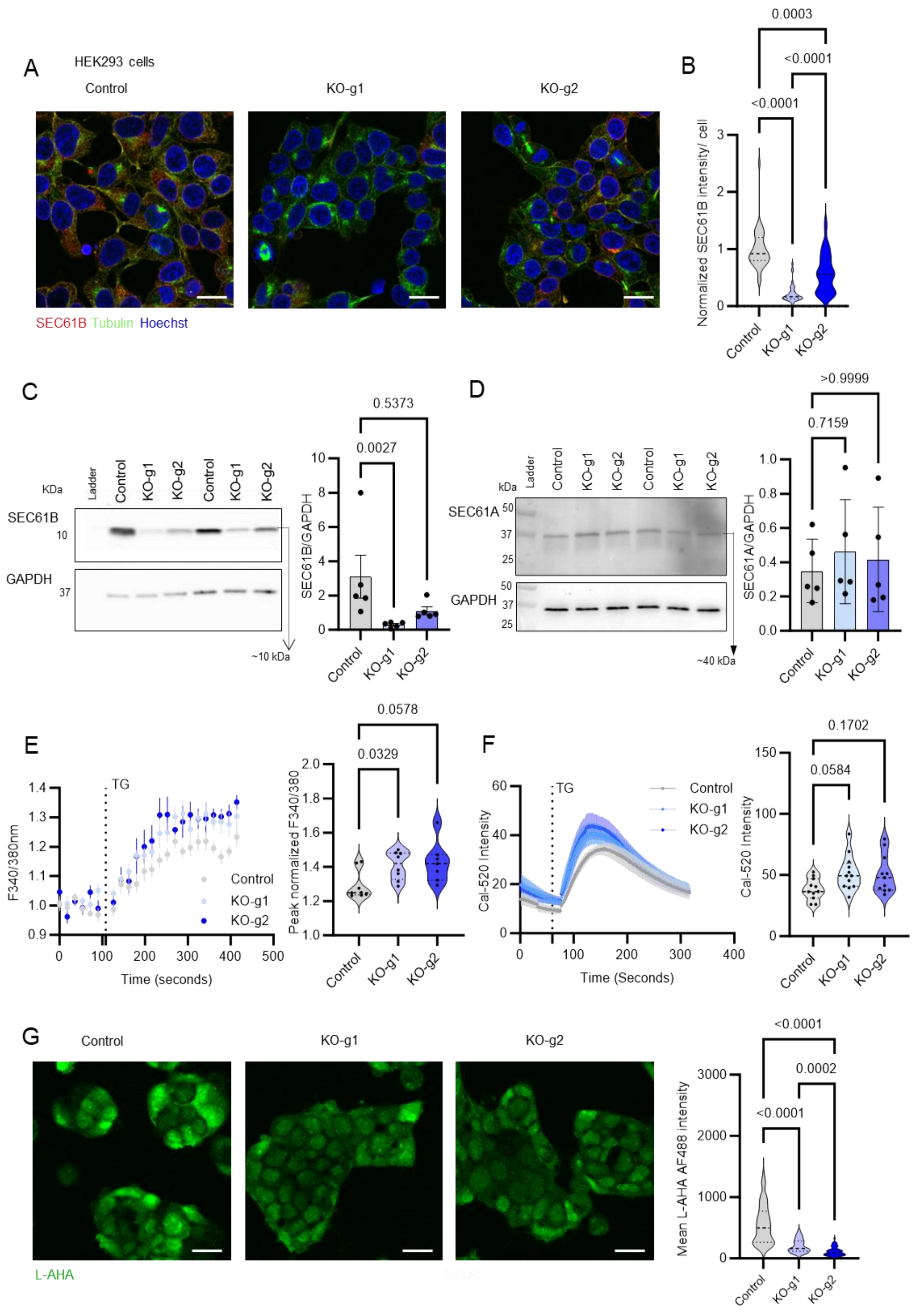

Supplementary Figure 5. Knockout of *SEC61B* leads to increased ER calcium leak and reduced protein synthesis. A. Representative images of SEC61B

expression (red) in control and CRISPR/Cas9-mediated *SEC61B* knockout (KO), using two guide RNAs (g1 and g2) in HEK293 cells. Tubulin stain is shown in green and nuclear staining with Hoechst in blue. Scale bar is 20 μm **B.** Normalized SEC1B intensity/cell in control (grey), KO-g1 (light blue) and KO-g2 (dark blue) HEK293 cells. N=30-40 cell clusters from n=3 independent experiments per genotype, Kruskal-Wallis test. **C.** Western blots of SEC61B in control, KO-g1 and KO-g2 cells. Band density ratio of SEC61B to GAPDH in control (grey), KO-g1 (light blue) and KO-g2 (dark blue) cells. N=5 separate cultures per genotype; one-way ANOVA with Dunn's multiple comparisons test. **D.** Western blots of SEC61A1 in control, KO-g1 and KO-g2 cells. Band density ratio of SEC61A1 to GAPDH in control (grey), KO-g1 (light blue) and KO-g2 (dark blue) cells. N=5 separate cultures per genotype, one-way ANOVA with Dunn's multiple comparisons test. **E.** Time course and maximum normalised cytosolic calcium concentrations measured by Fura2 (340/380 nm) in control (grey), KO-g1 (light blue) and KO-g2 (dark blue) cells treated with thapsigargin (TG) to elicit the SEC61-mediated ER calcium leak. N=7-8 independent experiments per genotype, one-way ANOVA with Dunn's multiple comparisons test. **F.** Time course and maximum cytosolic calcium measured by Cal-520, in the absence of probenecid, in control (grey), KO-g1 (light blue), and KO-g2 (dark blue) cells after addition of TG. N=11-12 separate cultures. per genotype, one-way ANOVA with Dunn's multiple comparisons test. **G.** Representative images of protein synthesis (green) in control, KO-g1 and KO-g2 HEK293 cells, scale bar 20μm. Quantification of protein synthesis in control (grey), KO-g1 (light blue) and KO-g2 (dark blue) cells. N=3 separate cultures per genotype, one-way ANOVA with Dunn's multiple comparisons.

Supplementary Figure 6

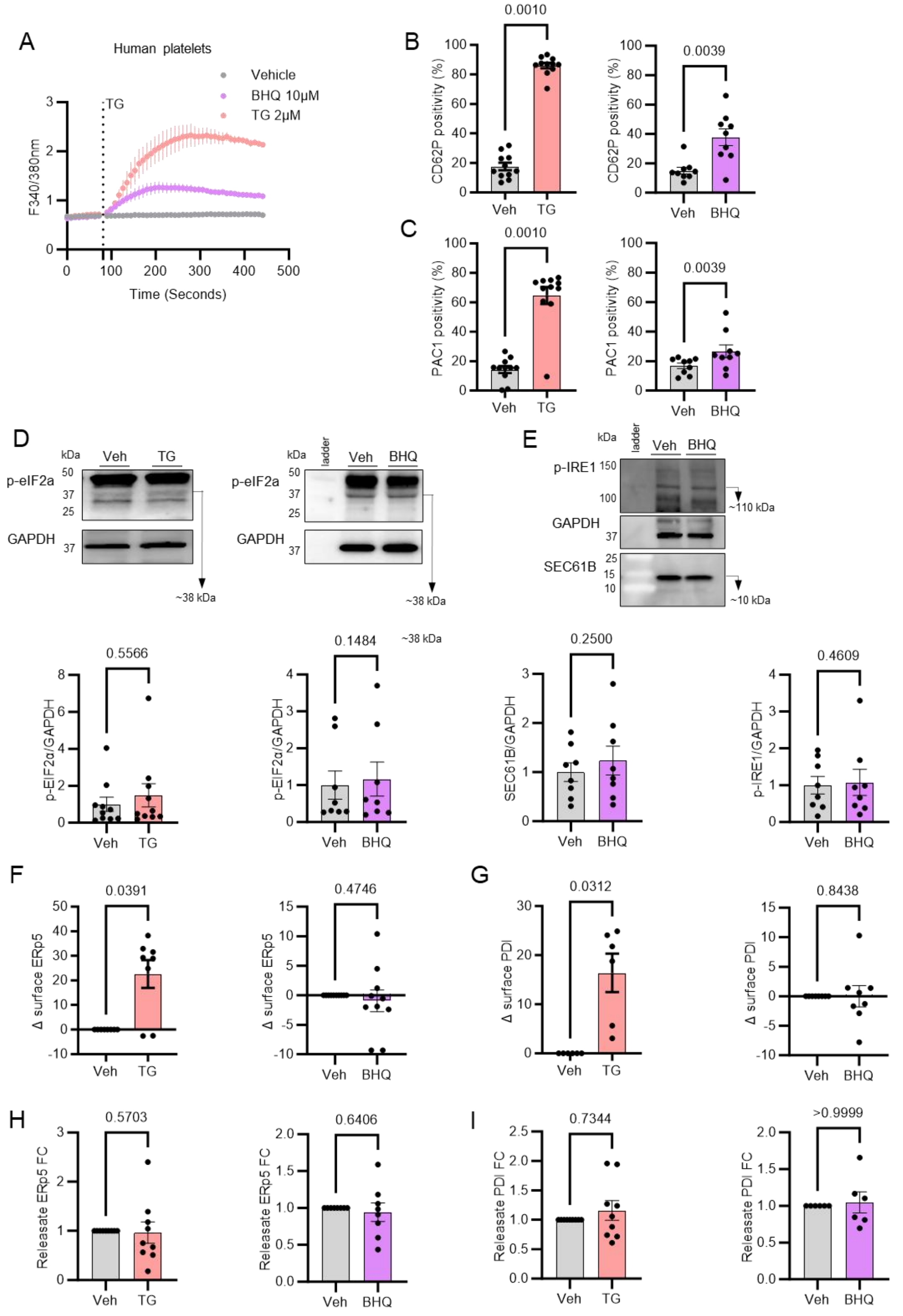

Supplementary Figure 6. ER calcium depletion is not associated with activation of the PERK pathway. Inhibition of SERCA3 does not upregulate SEC61B or activate the IRE1 pathway A. Time course of cytosolic calcium

changes after TG (2μM, red) (inhibitor of SERCA2, “strong”) and BHQ (10 μM, purple) (inhibitor of SERCA3, “moderate”) in human platelets. N=3 healthy donors. **B.** Percentage of CD62P and **C.** PAC-1 positivity of platelets treated with Veh (grey), TG (red), or BHQ (purple). **D.** Band density ratio of p-eIF2a to GAPDH by Western blot of platelets treated with Veh (grey), TG (red), or BHQ (purple). **E.** Band density ratio of SEC61B to GAPDH and p-IRE1 to GAPDH, by Western blot of platelets treated with Veh (grey), or BHQ (purple). p-IRE1 and SEC61B bands are in the same lane therefore sharing the same GAPDH control. **F.** Difference in platelet surface expression of ERp5 and **G.** PDI after treatment with TG (red), or BHQ (purple) compared with Veh (grey). **H.** Fold change of ERp5 and **I.** PDI in the platelet releasate after treatment with Veh (grey), TG (red), or BHQ (purple), as detected with Western blot. For Suppl figures 3B-3H data presented as mean ± SEM; n=8 healthy donors, Mann Whitney test. Veh: vehicle, BHQ: 2,5,-di-t-butyl-1,4-benzohydroquinone; TG: thapsigargin; p-eIF2a: phosphorylated eukaryotic translation initiation factor 2A, p-IRE1: phosphorylated inositol-requiring enzyme 1; Δ: difference; ERp5: endoplasmic reticulum protein 5; PDI: protein disulfide isomerase.
