# Supplementary material for: SEC61B regulates calcium flux and platelet hyperreactivity in diabetes
Source: J Clin Invest. 2025 Aug 15;135(16):e184597. doi: 10.1172/JCI184597 (PMC12352904; doi:10.1172/JCI184597)

Full unedited blot/gel for **Figure 2A**

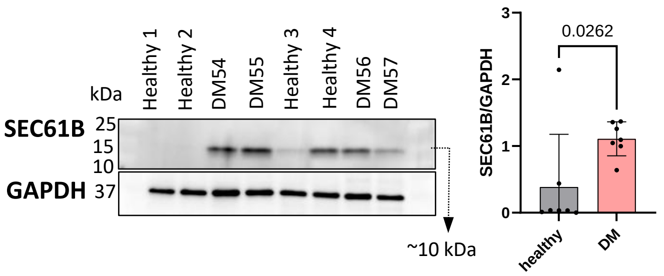

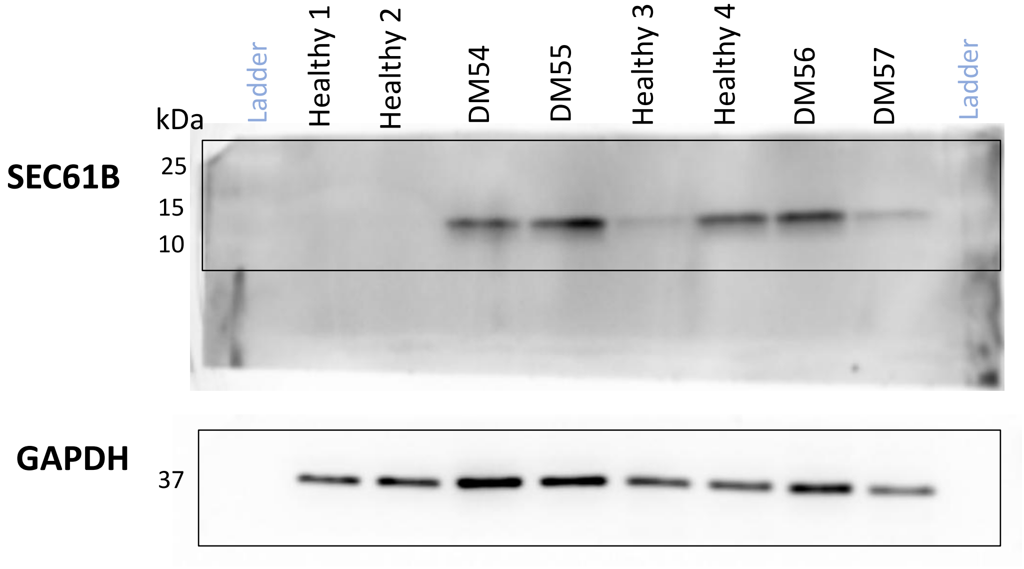

Full unedited blot/gel for **Figure 2B**

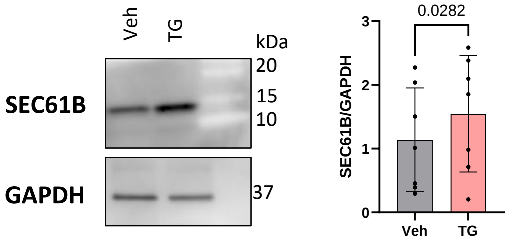

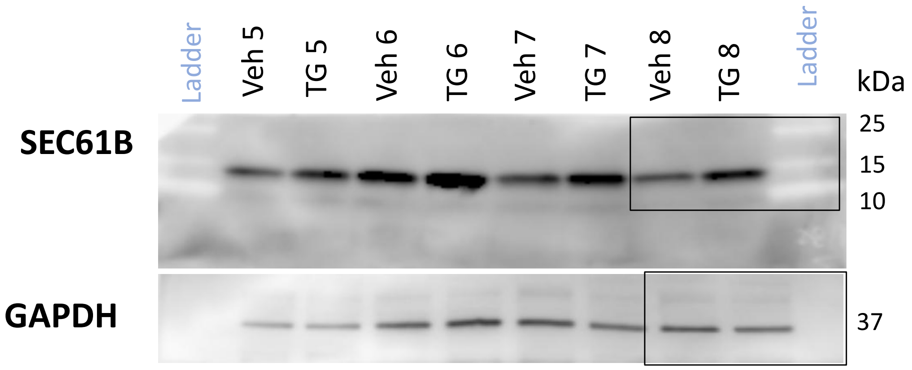

Full unedited blot/gel for **Figure 2C**

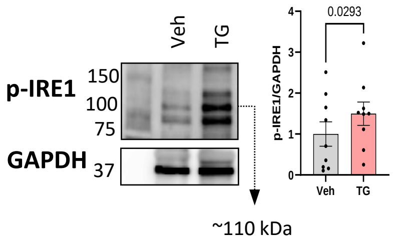

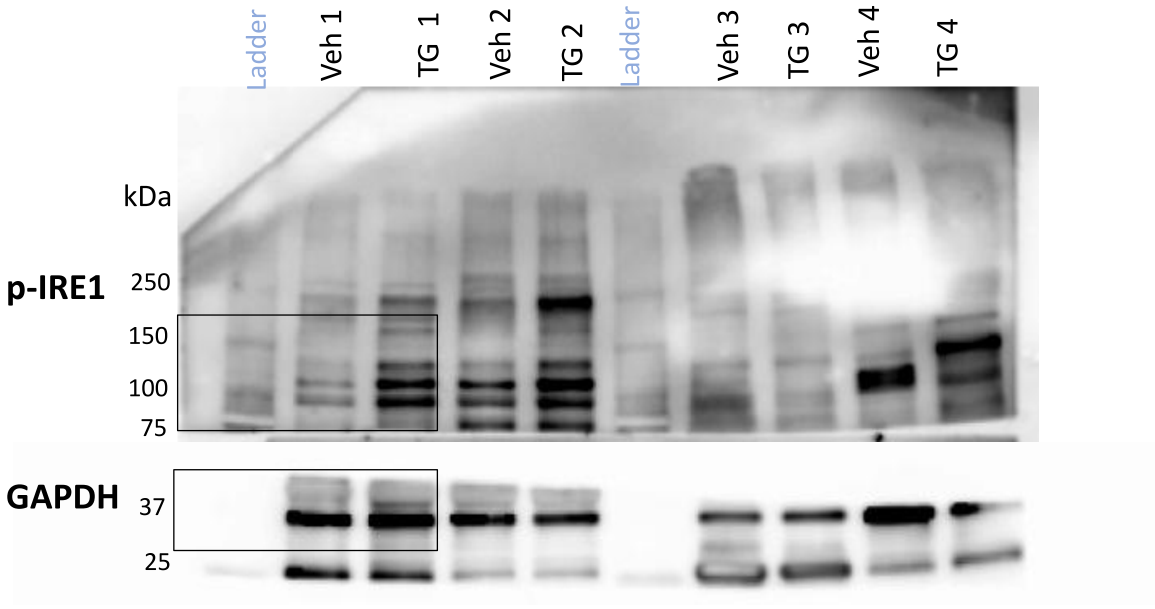

Full unedited blot/gel for **Figure 2E**

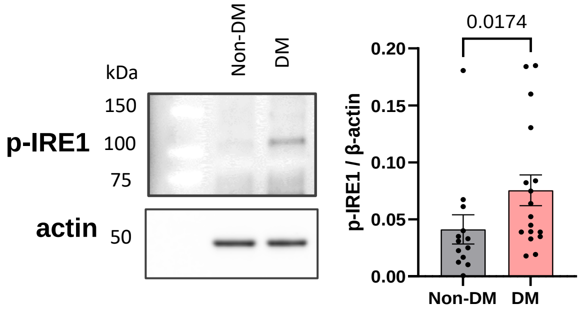

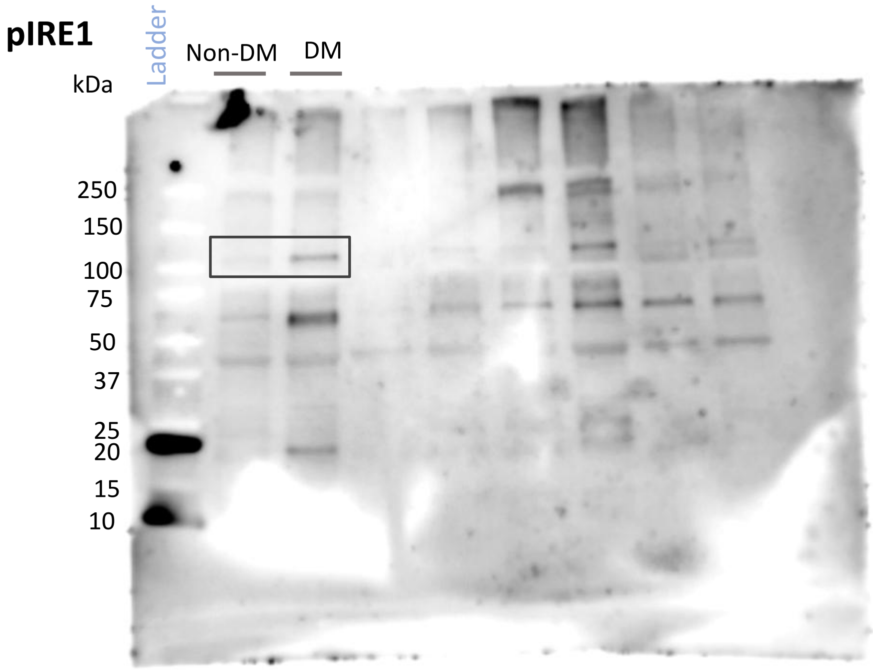

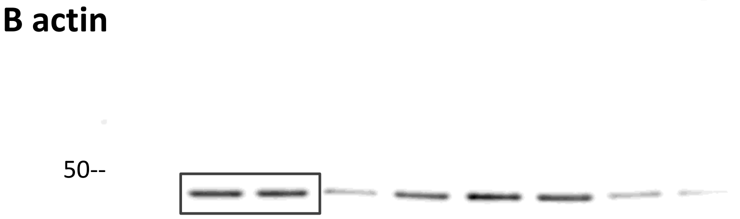

R2 Full unedited blot/gel for **Figure 2F**

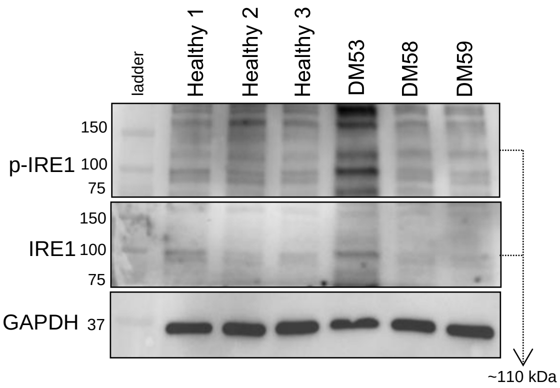

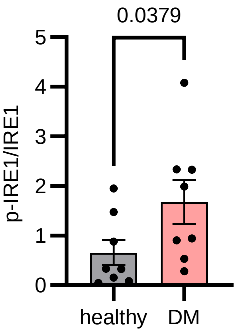

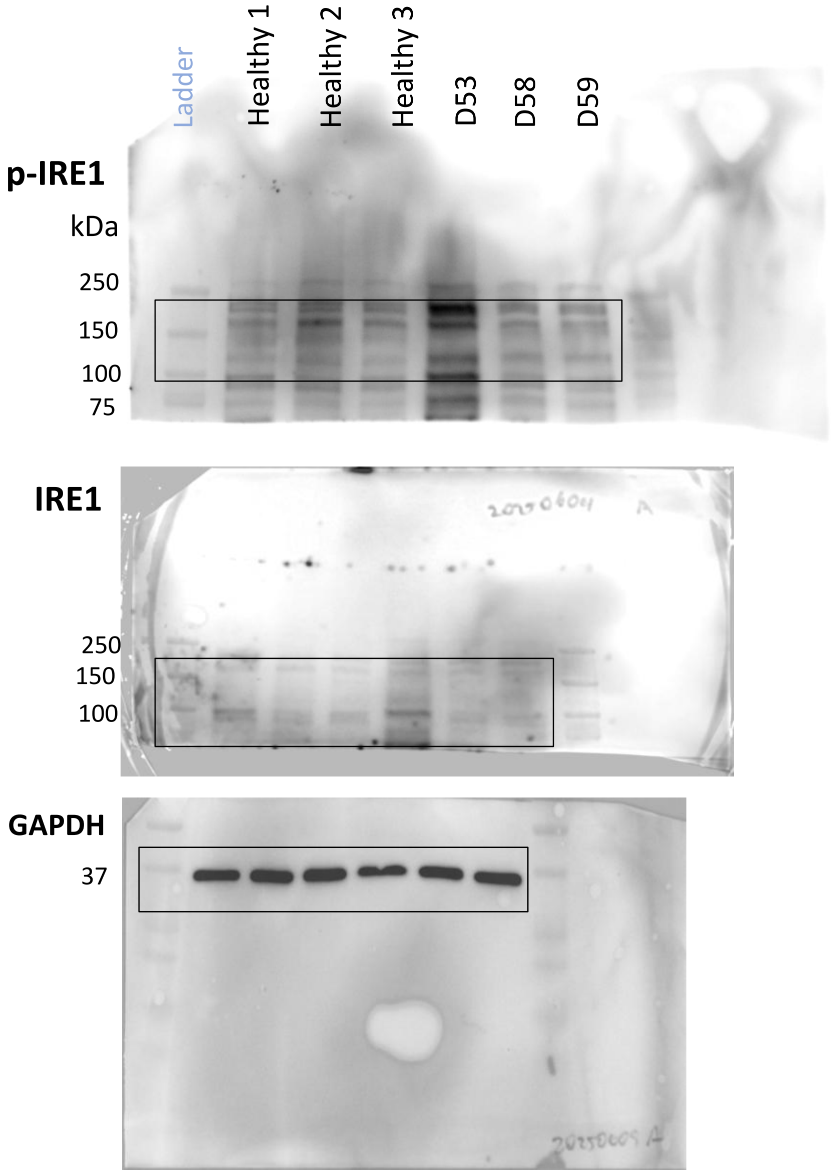

Full unedited blot/gel for **Figure 3C**

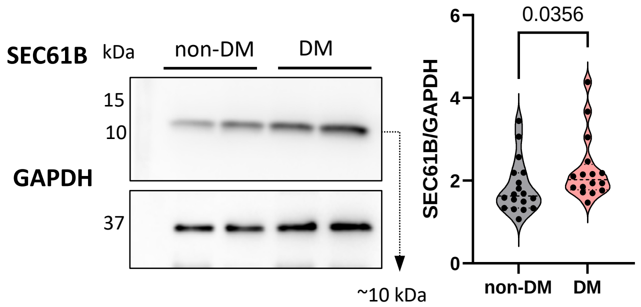

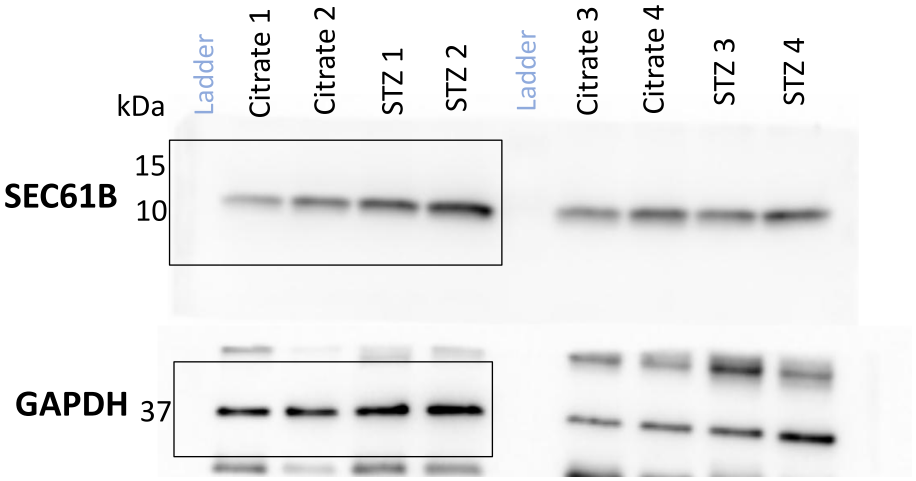

STZ = streptozotocin injected mice (DM)
Citrate= vehicle injected mice (non-DM)

Full unedited blot/gel for **Figure 3E**

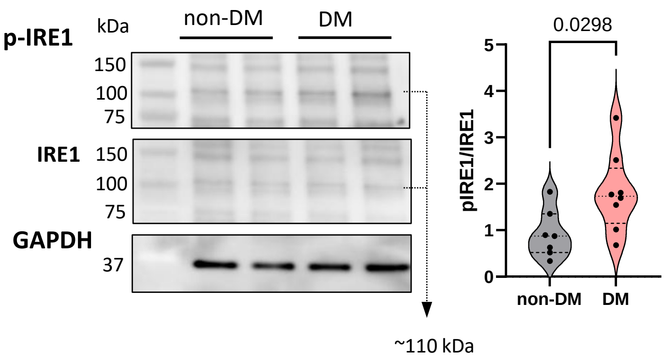

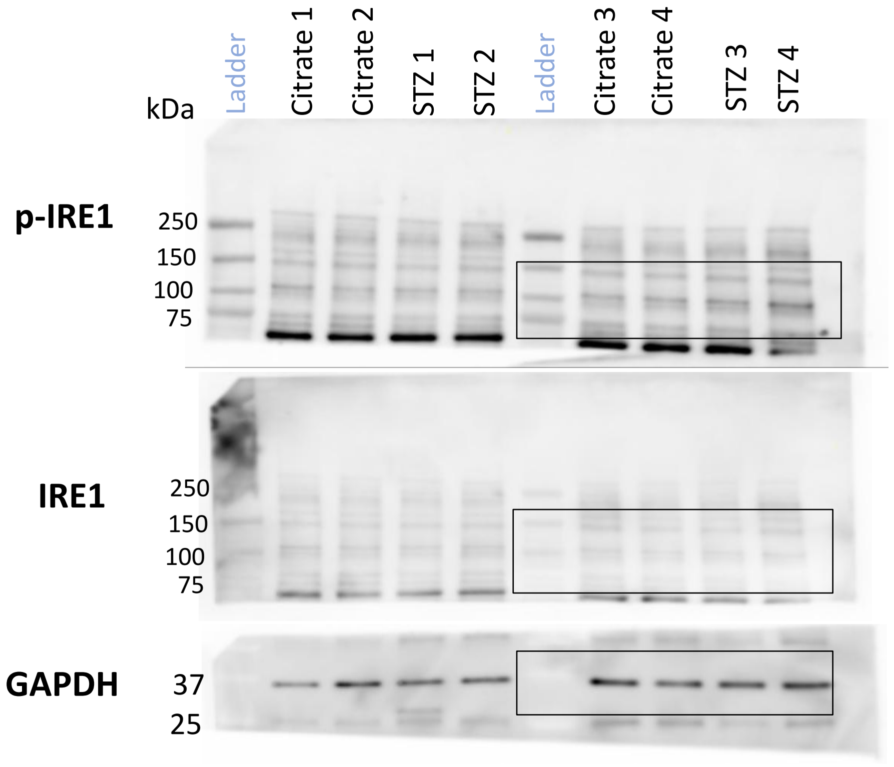

STZ = streptozotocin injected mice (DM)
Citrate= vehicle injected mice (non-DM)

Full unedited blot/gel for **Figure 5B**

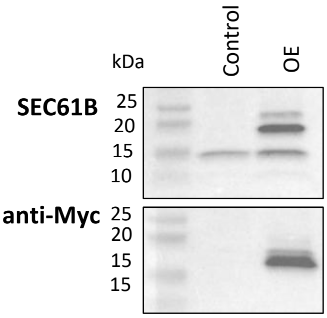

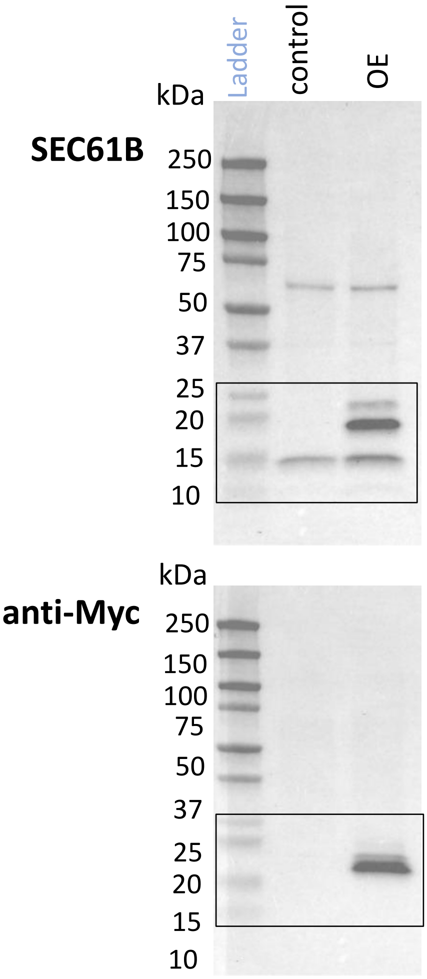

Full unedited blot/gel for **Figure 5C**

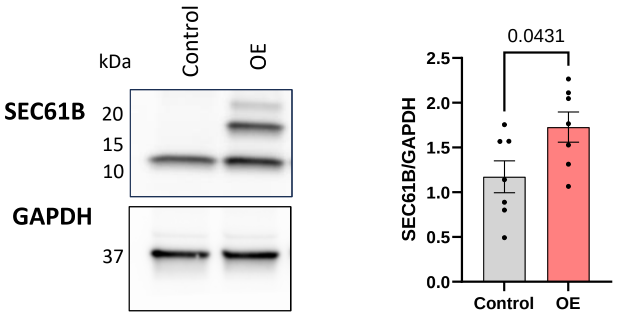

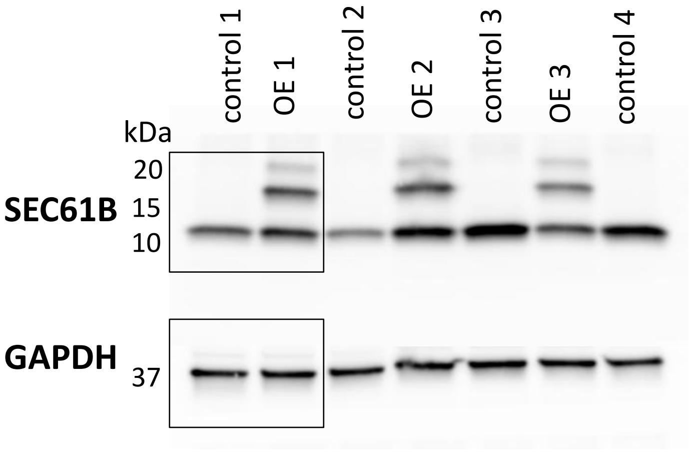

Full unedited blot/gel for **Figure 5D**

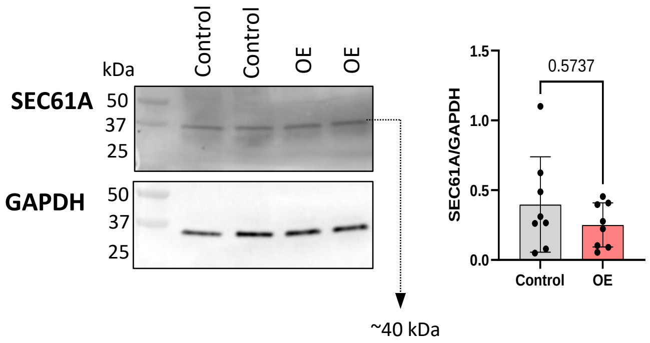

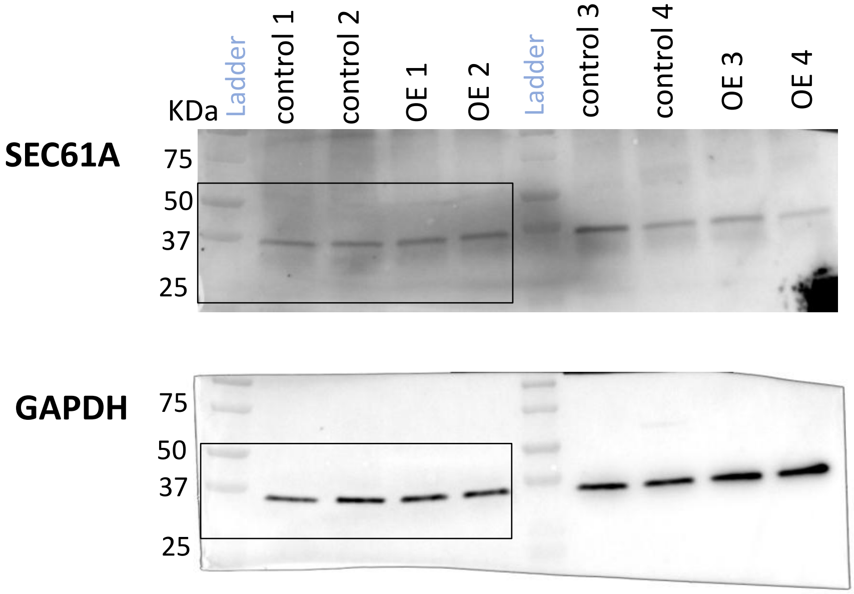

Full unedited blot/gel for **Figure 5E**

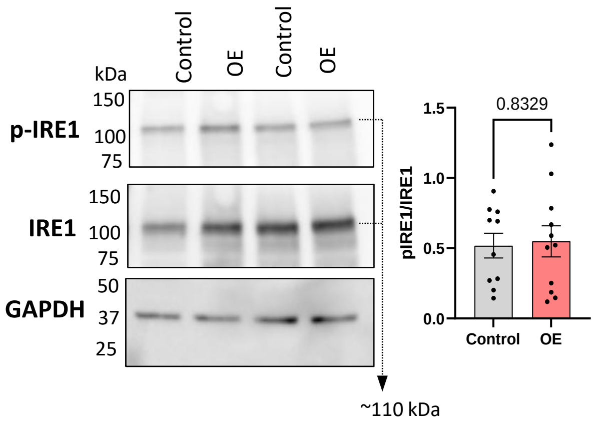

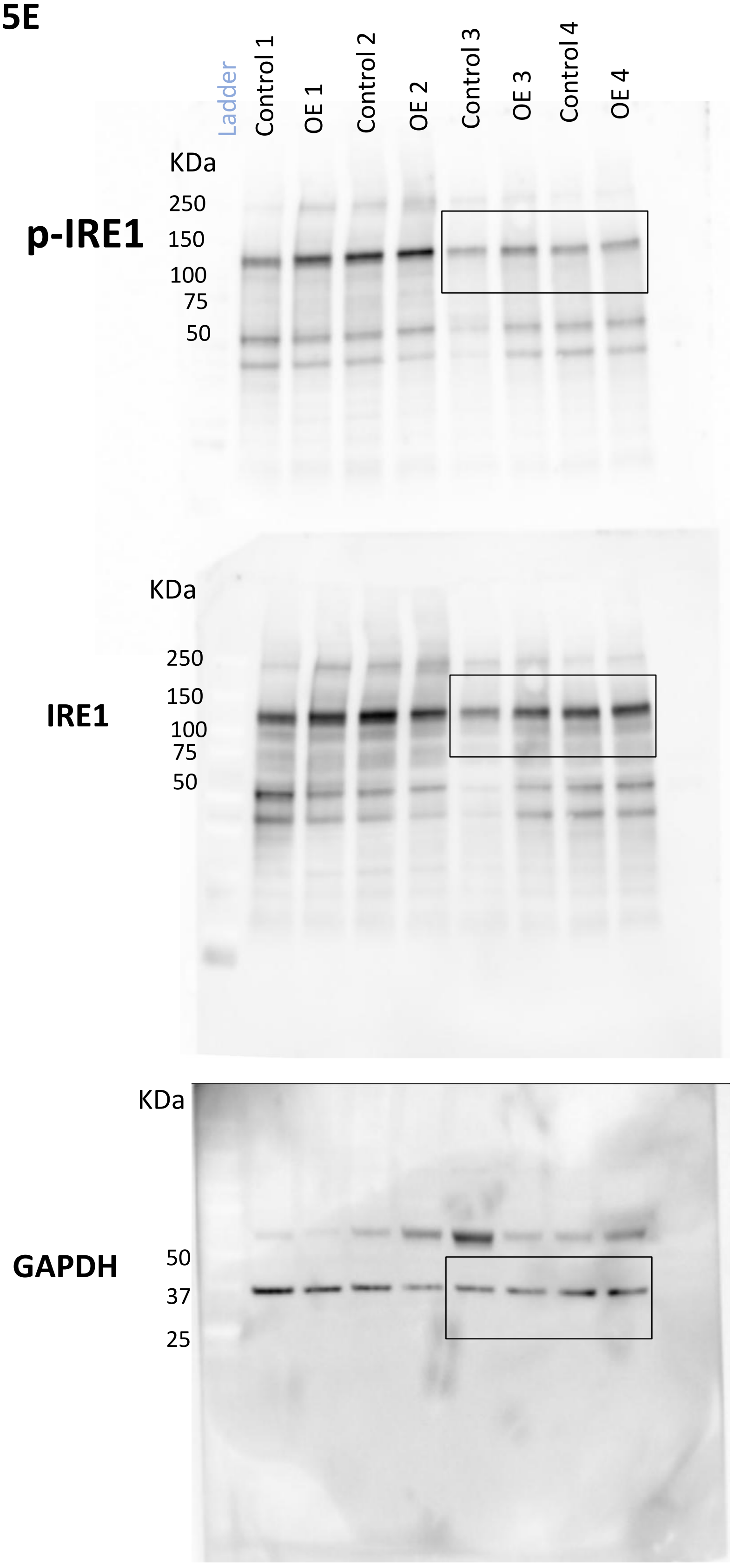

Full unedited blot/gel for **Supplementary Figure 2B**

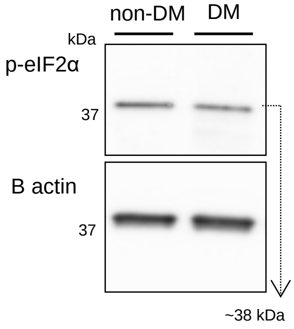

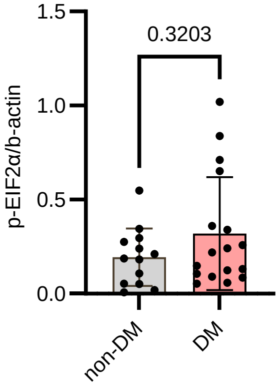

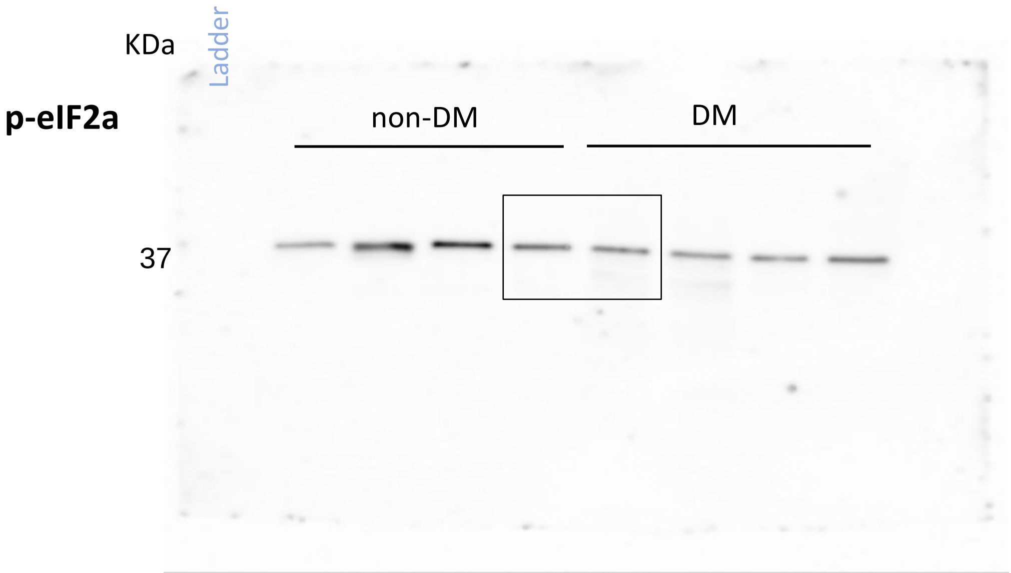

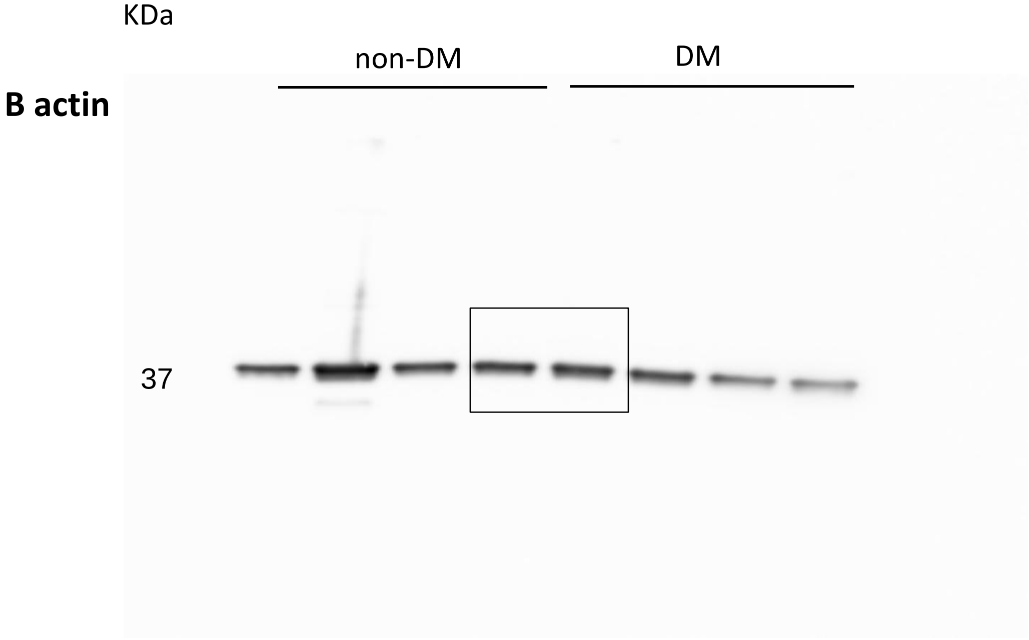

Full unedited blot/gel for **Supplementary Figure 4A**

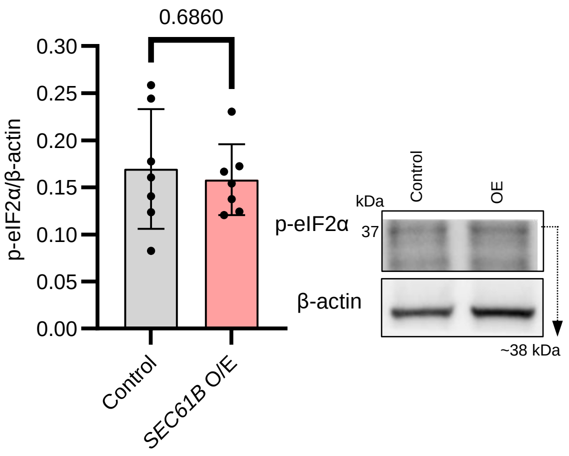

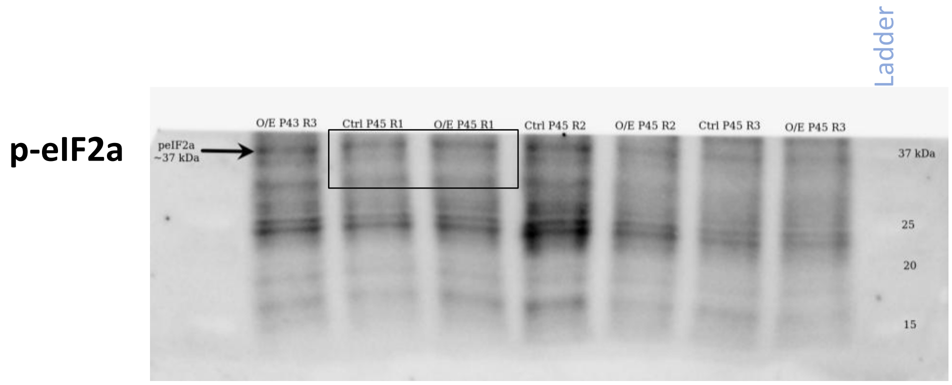

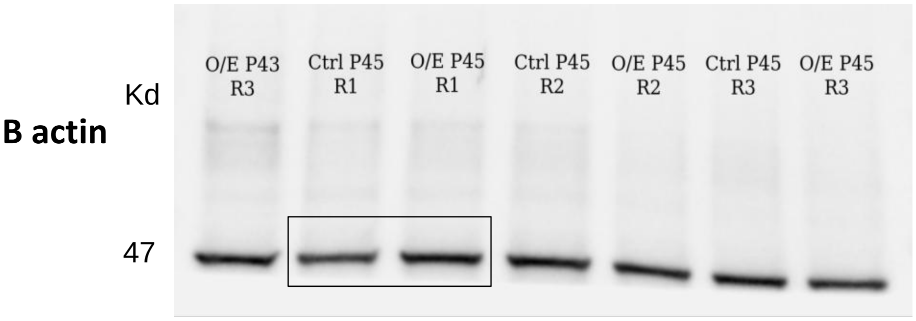

Full unedited blot/gel for **Supplementary Figure 4B**

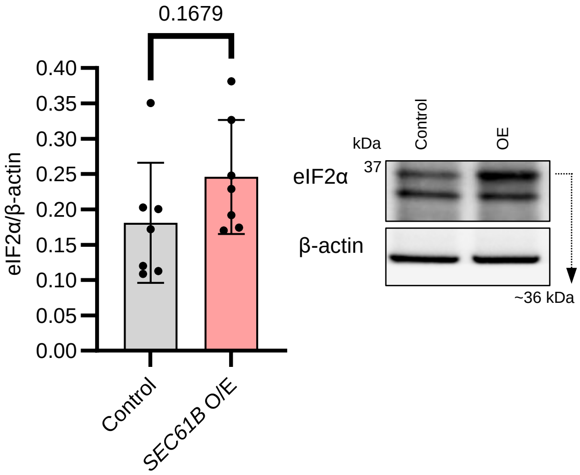

eIF2a

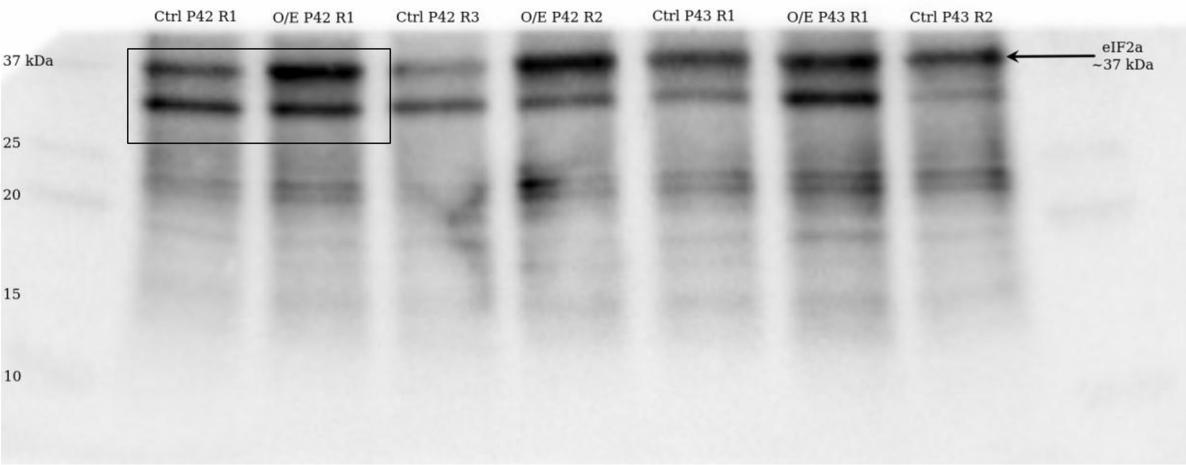

B actin

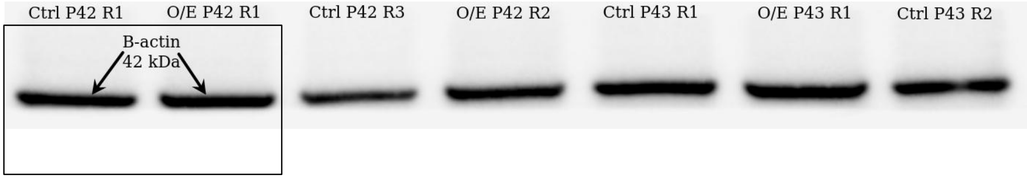

Full unedited blot/gel for **Supplementary Figure 4C**

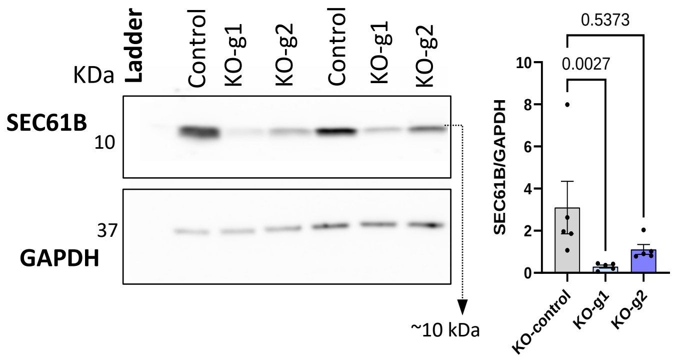

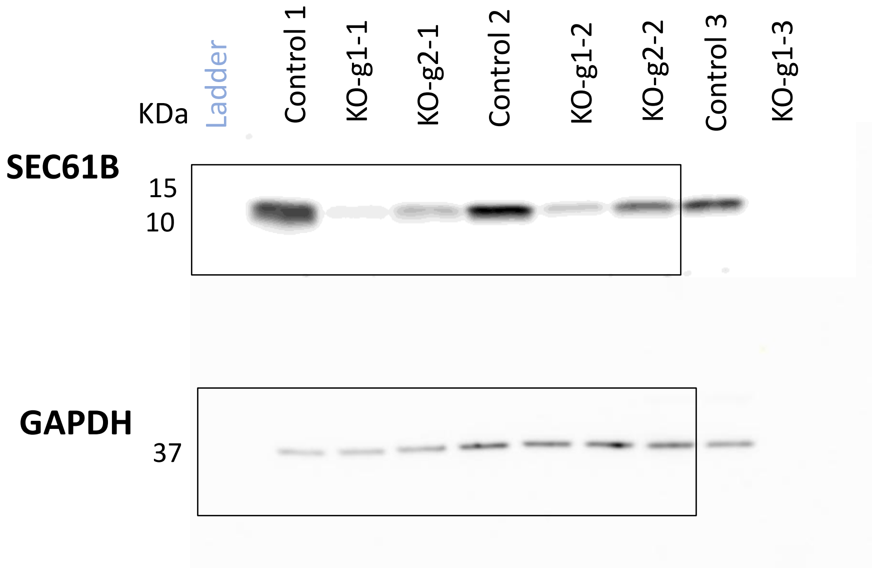

Full unedited blot/gel for **Supplementary Figure 4D**

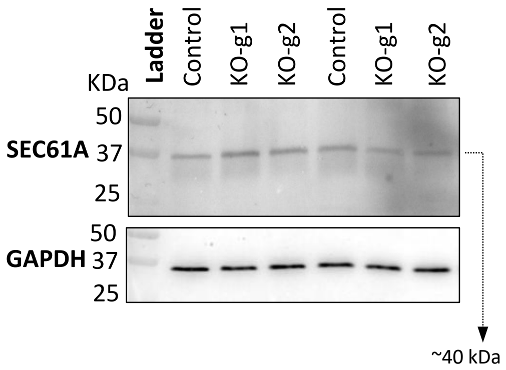

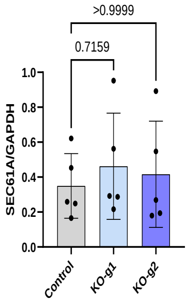

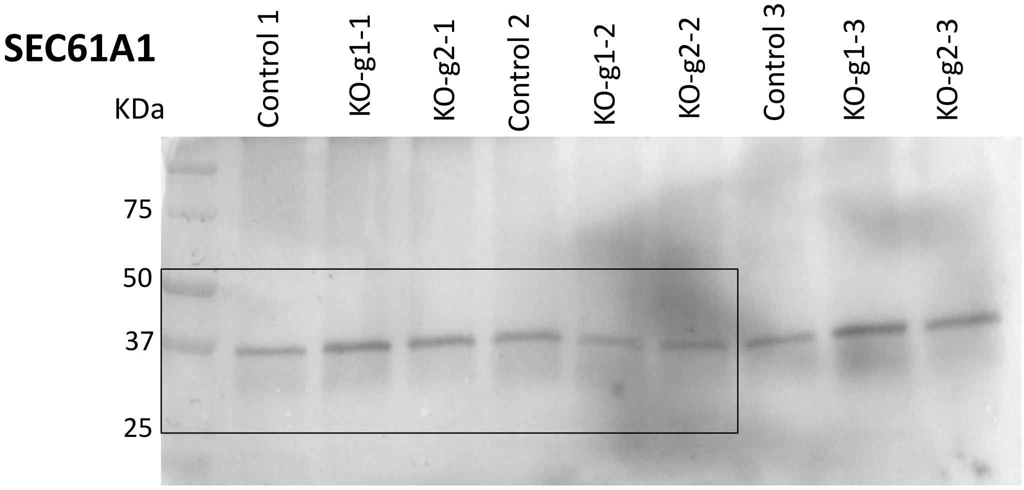

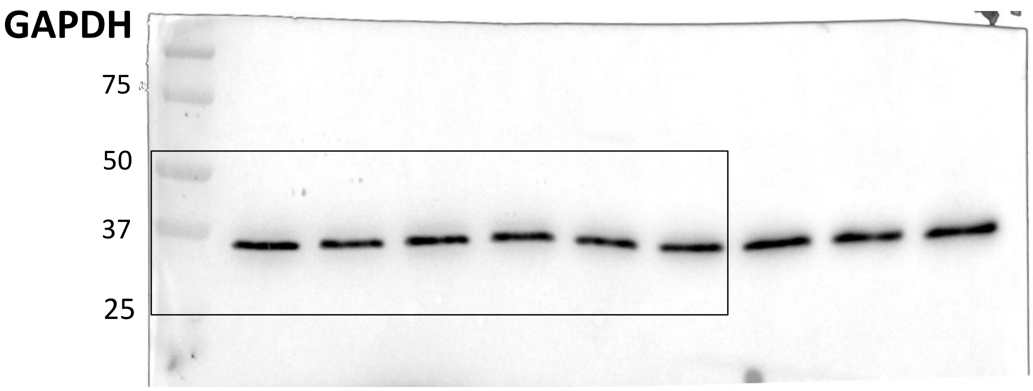

Full unedited blot/gel for **Supplementary Figure 6D**

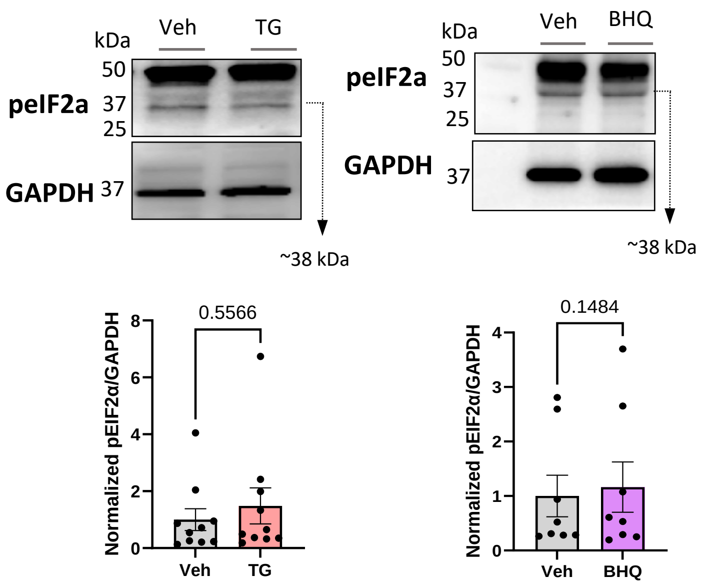

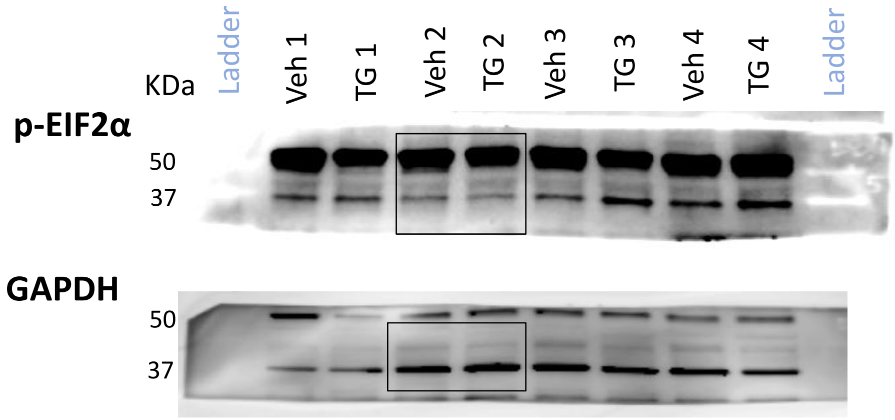

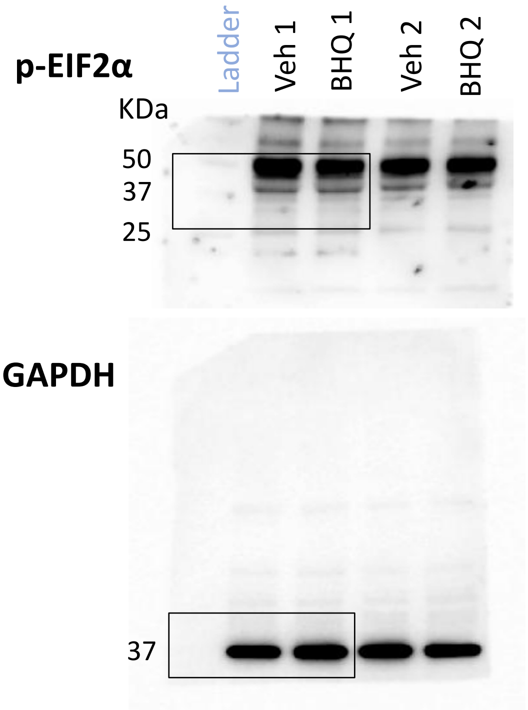

R2 Full unedited blot/gel for **Supplementary Figure 6E**

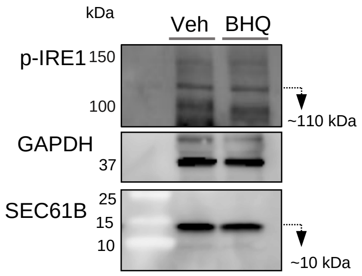

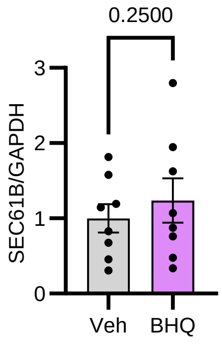

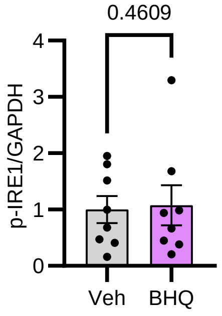

p-IRE1

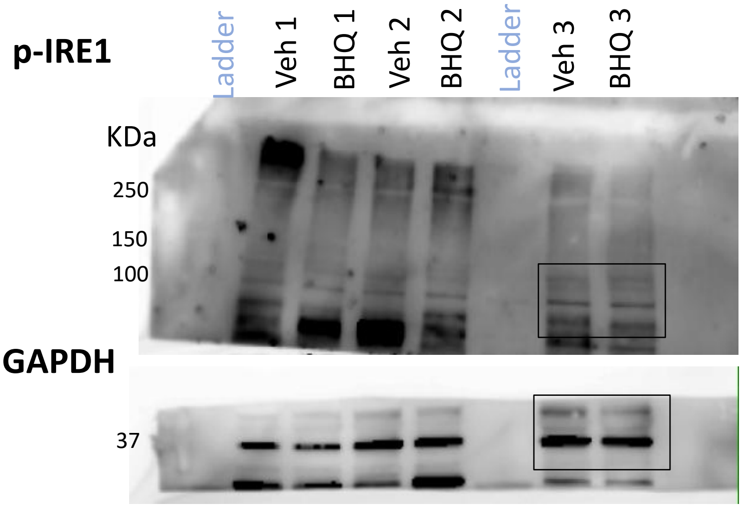

SEC61B

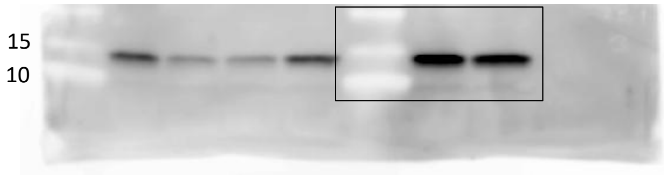

Supplement: Unedited blot and gel images [file jci-135-184597-s272.pdf]
